# Information Transmission in Delay-Coupled Neuronal Circuits in the Presence of a Relay Population

**DOI:** 10.3389/fnsys.2021.705371

**Published:** 2021-07-29

**Authors:** Jaime Sánchez-Claros, Aref Pariz, Alireza Valizadeh, Santiago Canals, Claudio R. Mirasso

**Affiliations:** ^1^Instituto de Física Interdisciplinar y Sistemas Complejos (IFISC, UIB-CSIC), Campus UIB, Palma de Mallorca, Spain; ^2^Institute for Advanced Studies in Basic Sciences, Zanjan, Iran; ^3^Department of Biology, University of Ottawa, Ottawa, ON, Canada; ^4^Instituto de Neurociencias, Consejo Superior de Investigaciones Científicas, Universidad Miguel Hernández, Sant Joan d'Alacant, Spain

**Keywords:** delay-coupled neuronal circuits, information transmission, synchronization, V and circular motifs, feature binding problem, thalamo-cortical circuit

## Abstract

Synchronization between neuronal populations is hypothesized to play a crucial role in the communication between brain networks. The binding of features, or the association of computations occurring in spatially segregated areas, is supposed to take place when a stable synchronization between cortical areas occurs. While a direct cortico-cortical connection typically fails to support this mechanism, the participation of a third area, a relay element, mediating in the communication was proposed to overcome this limitation. Among the different structures that could play the role of coordination during the binding process, the thalamus is the best placed region to carry out this task. In this paper we study how information flows in a canonical motif that mimics a cortico-thalamo-cortical circuit composed by three mutually coupled neuronal populations (also called the V-motif). Through extensive numerical simulations, we found that the amount of information transferred between the oscillating neuronal populations is determined by the delay in their connections and the mismatch in their oscillation frequencies (detuning). While the transmission from a cortical population is mostly restricted to positive detuning, transmission from the relay (thalamic) population to the cortical populations is robust for a broad range of detuning values, including negative values, while permitting feedback communication from the cortex at high frequencies, thus supporting robust bottom up and top down interaction. In this case, a strong feedback transmission between the cortex to thalamus supports the possibility of robust bottom-up and top-down interactions in this motif. Interestingly, adding a cortico-cortical bidirectional connection to the V-motif (C-motif) expands the dynamics of the system with distinct operation modes. While overall transmission efficiency is decreased, new communication channels establish cortico-thalamo-cortical association loops. Switching between operation modes depends on the synaptic strength of the cortico-cortical connections. Our results support a role of the transthalamic V-motif in the binding of spatially segregated cortical computations, and suggest an important regulatory role of the direct cortico-cortical connection.

## 1. Introduction

The synchronization of neuronal populations is a ubiquitous phenomenon in the brain circuits. The correlated activity of neurons in local networks gives rise to the appearance of brain oscillations over different frequency ranges (Başar et al., 2000; Pfurtscheller et al., 2000; Başar, 2012; Jensen et al., 2019). Based on the coherence and phase relations between oscillations in different brain regions, it has been proposed that information transmission and even directionality can be modulated (Eckhorn et al., 1988; Pfurtscheller et al., 2000; Gross et al., 2001; Fries, 2015; Maris et al., 2016). As a consequence, coherent activity in brain networks is hypothesized to underlie several cognitive phenomena such as features binding (Singer, 2007; Opitz, 2010; Coll et al., 2018), attention (Borisyuk et al., 1999; Niebur, 2002; Doesburg et al., 2008), working memory (Baddeley, 1992, 2010; Baddeley and Hitch, 2010) and motor function (Feige et al., 2000; Baker et al., 2003; Denker et al., 2007), among others.

An interesting case of the synchrony between different brain regions is the zero-lag synchronization, which can be observed even between distant cortical areas (Chawla et al., 2001; Vicente et al., 2008; Viriyopase et al., 2012; Esfahani and Valizadeh, 2014; Gollo et al., 2014), representing a suitable mechanism for the binding of sensory features into integrated and coherent perception. This phenomenon has been subject of controversial debate for many years: how two distant brain areas can synchronize at (almost) zero-lag despite the presence of non-negligible delays in their connections (Vicente et al., 2008; Viriyopase et al., 2012). However, a relatively simple and widespread motif found in neural circuits, a chain of three mutually delay-coupled oscillatory populations has been shown to support zero-lag synchronization (Fischer et al., 2006; Uhlhaas et al., 2009; Gollo et al., 2014). The biological relevance of such connectivity pattern, usually called V-motif, is justified for instance in the cortico-thalamic loops. The V-motif can determine a cortico-thalamo-cortical circuit in which the thalamus plays the role of the intermediate element (higher order relay) indirectly connecting two cortical regions (Save and Poucet, 2000; Uhlhaas et al., 2009; Sherman, 2012; Sysoeva et al., 2016). The V-motif circuit attracted much interest in recent years and several studies have been devoted to the exploration of its dynamical properties, either considering single neurons/oscillators or neural populations (Sporns and Kötter, 2004; Fischer et al., 2006; Esfahani and Valizadeh, 2014; Mirasso et al., 2017).

The addition of a cortico-cortical connection, that is known to play an important role in cortical circuits, transforms the V-motif into a circular motif (C-motif). In fact, this extension of the V-motif finds a counterpart in biological cortico-thalamic circuits (Sherman, 2016). Indeed, interaction between the direct cortico-cortical and the indirect transthalamic pathways, which converge onto individual postsynaptic cells in layer 4 of the cortex (Lee and Sherman, 2008; De Pasquale and Sherman, 2011), is hypothesized to have an important role in information transfer between areas (Sherman, 2016).

Although the structure of the adult brain does not change in short time scales, the efficacy of the synaptic connections and the excitation/inhibition balance are subject to continuous change and can evolve almost instantaneously, enabling the brain to flexibly exploit the fixed structure in a multiplex of tasks (Friston, 1994, 2011; Deco et al., 2008; Hutchison et al., 2013; Avena-Koenigsberger et al., 2018) According to communication through coherence (CTC) theory, the synchrony and phase relationship between neuronal populations can modulate the effective connectivity between brain areas and the direction of the information transfer in brain circuits (Fries, 2015). This means that a change in the phase difference due to changes in the network parameters affects the effective connectivity, a fact that has been shown in several computational studies in two-component networks (Barardi et al., 2014; Sancristóbal et al., 2014; Kirst et al., 2016; Palmigiano et al., 2017; Pariz et al., 2018, 2021). It is natural then to explore the effective connectivity in the proposed cortico-thalamic-cortical circuit since its structure influences the phase relationships between the regions. It is known that the arrangement of the three neural populations in a V-motif favors the state of zero-lag synchronization between outer populations. However, the effective connectivity in this network is poorly understood and we did not find studies that address how information is transmitted in this neural population network, as well as in other three-component networks. In fact, most studies of functional connectivity have focused on two-component motifs, as mentioned before, and the consequences of pairwise communication on patterns transferred in larger networks (Barardi et al., 2014; Sancristóbal et al., 2014; Kirst et al., 2016; Palmigiano et al., 2017; Pariz et al., 2018, 2021). Since the CTC theory predicts the modulation of the effective connectivity due to changes in the phase relationships, we wonder how communication is affected in the V-motif, as well as in the C-motif, due to frequency detuning and communication delays.

In this study, we address the previous question by considering a network of three mutually delay-coupled neural populations arranged in a V-motif. We further analyze how a direct and reciprocal cortico-cortical connection affects on our results. We systematically explored the effect that a change in the connections delay and detuning between the natural oscillation frequencies of the populations have in the transmission of signals in these particular motifs. Our results show in the case of the V-motif that for small delays, an efficient transmission is achieved when the sender population oscillates faster than the receiver ones, in a good agreement with previous results (Barardi et al., 2014; Sancristóbal et al., 2014; Kirst et al., 2016; Pariz et al., 2018, 2021). For intermediate delays and when the sender population is the relaying node, a good transmission quality is obtained even if its oscillation frequency is slower than that of the receiver. However, the picture drastically changes when the signal is added to one of the outer populations. In this case, an efficient transmission only happens, for any delay, for positive values of the detuning, i.e., when the sender population oscillates with a higher frequency than the others. We discuss these results in the context of the known anatomy of the cortico-thalamo-cortical loop and its hypothesized functions.

The paper is organized as follows. In section 2 we present the material and methods used to model our system. In section 3 we describe the tools we used to analyze the results obtained, which are presented in detail in section 4. Finally, in section 5 we discuss some of the main results and highlight some conclusions.

## 2. Materials and Methods

### 2.1. Neural Model

Our neural population model is similar to the one used in Pariz et al. (2018) which employs the Hodgkin-Huxley neuron model (Hodgkin and Huxley, 1952). This model describes the evolution of the membrane potential and the gate variables as follows

(1) $$\begin{array}{l} C\frac{dv}{dt}=-g_{K}n^{4}\left(v-E_{k}\right)-g_{Na}m^{3}h\left(v-E_{Na}\right)\\ \;-g_{L}\left(v-E_{L}\right)+I_{\text{ext}}+I_{\text{syn}}+I_{\text{noise}},\\ \;\frac{dn}{dt}=\alpha_{n}\left(v\right)\left(1-n\right)-\beta_{n}\left(v\right),\\ \;\frac{dm}{dt}=\alpha_{m}\left(v\right)\left(1-m\right)-\beta_{m}\left(v\right),\\ \;\frac{dh}{dt}=\alpha_{h}\left(v\right)\left(1-h\right)-\beta_{h}\left(v\right). \end{array}$$

The functions α_*x*_ and β_*x*_, *x* = *n, m, h*, are define as

(2) $$\begin{array}{l} \;\alpha_{n}\left(v\right)=\frac{\left(v+55\right)/100}{1-\exp\left(-\left(v+55\right)/100\right)},\\ \alpha_{m}\left(v\right)=\frac{\left(v+40\right)/10}{1-\exp\left(-\left(v+40\right)/100\right)},\\ \;\alpha_{h}\left(v\right)=0.07\exp\left(\frac{-\left(v+65\right)}{20}\right),\\ \;\beta_{n}\left(v\right)=\frac{1}{80}\exp\left(\frac{-\left(v+65\right)}{80}\right),\\ \beta_{m}\left(v\right)=4\exp\left(\frac{-\left(v+65\right)}{18}\right),\\ \;\beta_{h}\left(v\right)=\frac{1}{1+\exp\left(-\left(v+35\right)/10\right)}, \end{array}$$

where *I*_ext_, *I*_syn_ and *I*_noise_ are the injected input current, the synaptic current and the gaussian white noise current, respectively. The values the parameters used in our calculations are shown in Table 1 (Pariz et al., 2021).

**Table 1:** Parameters of the model.

**C**	**1 μF/cm^**2**^**	**Capacitance**
*g*_*K*_	36 μS/cm^2^	K conductance
*g*_*Na*_	120 μS/cm^2^	Na conductance
*g*_*L*_	0.3 μS/cm^2^	Leak conductance
*E*_*K*_	–77 mV	K reversal potential
*E*_*Na*_	50 mV	Na reversal potential
*E*_*L*_	–54.4 mV	Leak reversal potential
*E*_syn,*E*_	0 mV	Excitatory reversal potential
*E*_syn,*I*_	–80 mV	Inhibitory reversal potential
τ_*inter*_	0–14 ms	Inter population delay
τ_*intra*_	0.5 ms	Intra population delay
τ_*d*_	3 ms	Synaptic decay time
τ_*r*_	0.5 ms	Synaptic rise time
*I*_ext_	10–12 μA/cm^2^	Injected current
μ	0 μA/cm^2^	Median of the gaussian white noise
σ	0.5 μA/cm^2^	Standard Deviation of the gaussian white noise
*g*_*EE*_	3.75 μS/cm^2^	Synaptic weight: excitatory → excitatory
*g*_*EI*_	7.5 μS/cm^2^	Synaptic weight: excitatory → inhibitory
*g*_*IE*_	15 μS/cm^2^	Synaptic weight: inhibitory → excitatory
*g*_*II*_	15 μS/cm^2^	Synaptic weight: inhibitory → inhibitory

The synaptic current *I*_syn,*i*_ of the *i*-th post-synaptic neuron is modeled as

(3) $$\begin{array}{l} \;I_{\text{syn},i}=\underset{j}{\sum }g_{ij}S_{ij}\left(t\right)\left(v_{i}-E_{\text{syn},j}\right),\\ S_{ij}\left(t\right)=\frac{1}{A}\left[\exp\left(\frac{-\left(t-t_{j}^{*}-\tau_{ij}\right)}{\tau_{r}}\right)-\exp\left(\frac{-\left(t-t_{j}^{*}-\tau_{ij}\right)}{\tau_{d}}\right)\right],\\ \;A={\left(\frac{\tau_{r}}{\tau_{d}}\right)}^{\frac{\tau_{r}}{\tau_{d}-\tau_{r}}}-{\left(\frac{\tau_{r}}{\tau_{d}}\right)}^{\frac{\tau_{d}}{\tau_{d}-\tau_{r}}}, \end{array}$$

where *v*_*i*_ is the membrane potential of the post-synaptic neuron and *E*_syn,*j*_ is the reversal synaptic potential of the post-synaptic neuron. The dynamics of *S*_*ij*_ is described by a double-exponential function, modeling the efficacy of AMPA and GABA_a_ chemical synapses (Pariz et al., 2021). Variable $t_{j}^{*}$ represents the time at which the *j*-th pre-synaptic neuron spikes and τ_*ij*_ is the axonal delay between the pre- and post-synaptic neurons. The values of the synaptic parameters are also given in Table 1.

### 2.2. Population Architecture

**Figure 1:**
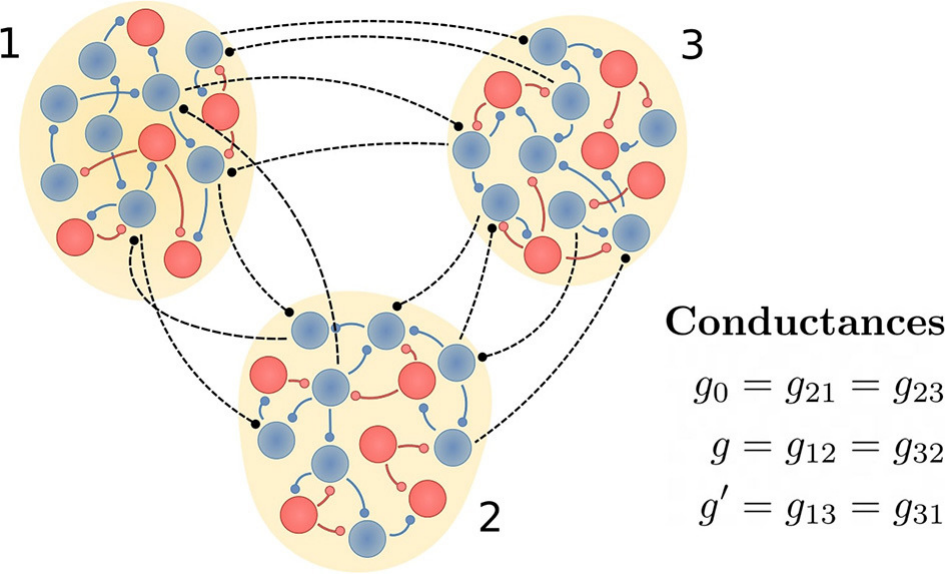
Schematic representation of the circuit. Neural populations 1 and 3 would represent cortical areas and the population 2 the thalamus. Excitatory and inhibitory neurons (as well as their synaptic projections) are shown in blue and red, respectively. Inter-population connections are only excitatory and, depending on the case of interest, their synaptic weights are modified. If the connections between populations 1 and 3 are absent, then we call it a V-motif circuit. Otherwise, the circuit is a circular motif (C-motif).

Each population is composed of 100 neurons described by the Hodgkin-Huxley equations, where 80 are considered as excitatory and 20 as inhibitory. Each neuron is randomly connected to 10% (5%) of neurons in the same (different) population. The intra-population delay is set to 0.5 ms and the inter-population delay τ is varied from 0 to 14 ms. While the intra-population connections are both excitatory and inhibitory, the inter-population connections are assumed to be only excitatory. In Figure 1 we schematically represent the three populations and their connections.

Each neuron receives an external constant injection current, bias current, that varies between 10 and 12 μA/cm^2^. Additionally, each neuron receives a Gaussian white noise current with zero mean (μ = 0 μA/cm^2^), and standard deviation σ = 0.5 μA/cm^2^. Since the level of coherency and synchronization is very high (Pariz et al., 2021) we can consider each population as a collective oscillator with a well-defined frequency. For the values of the parameters we have chosen and depending on the bias current, the oscillation frequency varies linearly from 68 to 73 Hz (see Supplementary Figure 1).

We define the detuning or the frequency mismatch as the difference of the natural oscillation frequency of an isolated population with respect to those of the other populations. In our simulations, we drive the sender by external current *I*, and the other two populations by *I*_0_ = 11 μA/cm^2^, where their difference Δ*I* = *I* − *I*_0_, sets the frequency detuning. When we apply a frequency mismatch to a certain population, we do it by changing the bias current of all the neurons in that population and keeping that of the neurons of the other populations fixed at *I*_0_. To measure the transmission of signals we apply two types of perturbations, a slow (5 Hz) aperiodic signal and a fast pulsating signal (~ 70 Hz).

### 2.3. Simulations

For the numerical implementation we used the Mil'shtein algorithm (Milshtein, 1975) with a time step Δ*t* = 0.01 ms. The total simulation time used to analyze the transmission quality of a signal was different depending on whether the applied modulation was slow or fast. We used 6 s for the case of the slow signal-modulation and 4.2 s for fast signals. We varied the inter-population delay τ between populations and the detuning or frequency mismatch Δν (or equivalently Δ*I*) of the sender population. All the simulations were performed in Brian simulator (Goodman and Brette, 2009) written in the Python programming language.

## 3. Analysis

Our analysis was done using an approximation of the firing rate *r*(*t*). We computed first the multi unit activity *s*(*t*) as the total number of spikes that occur between *t* and *t* + Δ*t*. For each neural population, the firing rate *r* was computed as follows (Dayan and Abbott, 2001),

(4) $$\begin{array}{l} r\left(t\right)=\frac{1}{N\Delta t}\int_{-\infty }^{\infty }s\left(t-t^{\prime }\right)w\left(t^{\prime }\right)dt^{\prime }, \end{array}$$

where *N* is the total number of neurons in the population. This integral is called linear filter, and the window function *w*(*t*) is called the filter kernel which is considered here as a Gaussian function. We used σ = 2 ms and σ = 100 ms when fast and slow signals are injected to the system, respectively. In the absence of an external signal, σ = 2 ms was also considered to compute the firing rate.

### 3.1. Phase Difference

The phase of each neural population was computed from its collective oscillatory firing rate by the interpolation

(5) $$\begin{array}{l} \theta \left(t\right)=2\pi \;\left(\frac{t-t_{\text{max},k}}{t_{\text{max},k+1}-t_{\text{max},k}}\right), \end{array}$$

for *t*_max,*k*_ ≤ *t* < *t*_max,k+1_, where {*t*_max,*k*_, *k* = 1, ..., *k*_max_} are the relative maxima of the time series. So *t*_max,*k*_ denotes the initial time of the *k*-th oscillation cycle of the firing rate. This definition works independently of the periodicity of the time series. This approximated phase adapts to possible variations in the cycle duration. The phase difference between two populations, *i* and *j*, is then given by

(6) $$\begin{array}{l} \theta_{ij}\left(t\right)=\text{mod}\left[\theta_{i}\left(t\right)-\theta_{j}\left(t\right),2\pi \right], \end{array}$$

which was further shifted to be defined in the interval [−π, π). Then, the representative phase difference between the oscillations of a given pair of populations was set to be the median of the time series θ_*ij*_(*t*).

### 3.2. Phase Locking

To quantify the phase locking between two populations, we estimated how the phase difference distribution {*p*_θ_*ij*_, *k*_} approximated the perfect locked distribution, *i.e*., a delta-Dirac function. To this end we computed the Bhattacharyya coefficient (Kailath, 1967). Given two discrete probability distributions {*q*_*k*_} and {*h*_*k*_} (*k* = 1, 2, …, *n*), this coefficient is defined by

(7) $$\begin{array}{l} B\left(\left\{q_{k}\right\},\left\{h_{k}\right\}\right)=\overset{n}{\underset{k=1}{\sum }}\sqrt{q_{k}h_{k}}. \end{array}$$

Note that when the two distributions are identical *B* = 1 while two orthogonal distributions have *B* = 0.

In our analysis, the perfect locked distribution was defined as

(8) $$\begin{array}{l} \delta_{k}=\{\begin{array}{ll} 1 & \text{if }k=k^{*}:\;p_{\theta_{ij},k^{*}}=\max_{k}\left(p_{\theta_{ij},k}\right)\\ 0 & \text{otherwise} \end{array} \end{array}$$

So, considering equation (8), we defined our phase-locking index as

(9) $$\begin{array}{l} D_{ij}=1-B\left(\left\{p_{\theta_{ij},k}\right\},\left\{\delta_{k}\right\}\right)=1-\sqrt{\underset{k}{\max}\left(\theta_{ij,k}\right)}. \end{array}$$

Therefore, perfect locking implies D = 0 and the maximum value is $1-1/\sqrt{n}$ when the distribution is uniform. This index allowed us to estimate the stable-unstable region frontiers. When D is approximately 0.35 phase-drift effects start to appear in the dynamics, and so, we used this value to delimit these frontiers.

### 3.3. Time-Delayed Mutual Information

For slow modulation, we measured the information shared by the populations considering their firing rates. Specifically, this information transfer was computed via the time-delayed mutual information dMI of populations *i* and *j* (Kirst et al., 2016; Pariz et al., 2021). The dMI considers the instantaneous firing rates *x*_*i*_(*t*) and the lagged firing rate *x*_*j*_(*t* + δ), being δ the time lag. The dMI expression is given by

(10) $$\begin{array}{l} {\text{dMI}}_{ij}\left(\delta \right)=\int \int p_{ij^{\left(\delta \right)}}\left(t\right)\log\left(\frac{p_{ij^{\left(\delta \right)}}\left(t\right)}{p_{i}\left(t\right)p_{j}\left(t\right)}\right)dx_{i}\left(t\right)dx_{j}\left(t+\delta \right), \end{array}$$

where *p*_*a*_(*t*) is the probability distribution of *x*_*a*_(*t*), with *a* = *i, j*, and $p_{i,j^{\left(\delta \right)}}\left(t\right)$ the joint distribution between *x*_*i*_(*t*) and *x*_*j*_(*t* + δ). Asymmetries in dMI_*i,j*_(δ) indicate a dominant direction in which the information is shared or transferred between the populations. Therefore, we quantified these asymmetries by using the difference

(11) $$\begin{array}{l} \Delta {\text{MI}}_{ij}={\text{MI}}_{i\to j}-{\text{MI}}_{j\to i},\\ \;{\text{MI}}_{i\to j}=\int_{0}^{\infty }{\text{dMI}}_{ij}\left(\delta \right)\text{d}\delta ,\\ \;{\text{MI}}_{j\to i}=\int_{-\infty }^{0}{\text{dMI}}_{ij}\left(\delta \right)\text{d}\delta . \end{array}$$

If ΔMI_*ij*_ is positive, the information is mainly transferred from the population *i* to *j*, while a negative value indicates the opposite direction. Additionally, the higher this quantity, the better the transmission, and vice versa.

The number of bins of the probability distributions were computed by the algorithm described in Hacine-Gharbi et al. (2012). The maximum lag we considered for computing the time-delayed mutual information was 200 ms which is the period of the slow modulation (5 Hz).

For each value of the difference ΔMI_*i,j*_ computed, we determined whether or not it is statistically significant. To check this, we obtained the null-hypothesis distribution (lack of functional coupling). We generated 5,000 surrogates by a permutation technique of the time-series to build this distribution. After that, ΔMI_*i,j*_ values whose *p*-values were more than 0.05 were removed as an indicative of no statistically significance. For plot representation, we further applied a clustering algorithm to remove outliers that appeared.

### 3.4. Cross-Covariance

The cross-covariance quantifies the similarity between two time series as a function of the relative time distance between them. We used this quantity to determine the similarity between the firing rate of the receiving population with the external slow modulation. We assumed that the quality of the transmission is better if the firing rate follows the signal. We considered the non-normalized zero-lag cross-covariance (ZLC) to also detect differences in amplitude between the signals.

### 3.5. Phase Response Curve of the Population

When we considered the transmission of a fast signal, we used the phase response curve of the oscillating populations to quantify the quality of the transmitted signal. The phase response curve (PRC) is defined as the phase shift of an oscillation resulting from the application of an external perturbation with respect to the unperturbed case, as a function of the time at which the perturbation is applied (Ko and Ermentrout, 2009). This quantity is usually defined considering an isolated oscillator. However, in our system, each population (a collective oscillator) is coupled to one or two populations. To measure the response of a population to an injected pulse, we should proceed in a different way as it is usually done in the isolated case. Recent studies have proposed to measure information directionality based on the PRCs in a circuit of two mutually coupled oscillators under the weak coupling condition (Dumont and Gutkin, 2019).

The effect of an external perturbation in the sender population is quantified by the local phase response curve (lPRC), while that in the receiver populations is characterized by the non-local phase response curve (nPRC). The nPRC is defined as the response of an oscillator to a non-local perturbation (Schultheiss, 2012; Pariz et al., 2021). The process of finding the nPRC is similar to the PRC process, first we compute the positions of the peaks of the firing rate of all populations before applying the perturbation. Then, the perturbation pulse is applied at different phases (β) on all excitatory neurons of the first population, producing a change in the period of the oscillations. The effect of these phase changes, will propagate to the other populations, affecting the period of their oscillations. An important aspect is the axonal delay time between the populations. The response of the first population to the applied pulse will take an axonal delay time (τ) to reach the second population. By subtracting the periods of oscillation of the second and third populations, with and without the injected perturbation on the first population, gives the nPRC. In our study, we went one step further. Instead of applying the pulse at different phases and repeating the simulation to find the nPRC, we distributed the phase of the injected pulse along the oscillating period of the receiver population, considering the perturbation as a fast applied signal. The width of each pulse was taken as 2 ms and the amplitude as 0.25 μA/cm^2^.

We then quantified the information transmission *Z*_*i*_ as

(12) $$\begin{array}{l} Z_{i}=\int_{0}^{2\pi }|{\text{nPRC}}_{i}\left(\beta \right)|d\beta , \end{array}$$

where the nPRC measurements were first fitted to a 4th-order Fourier series function.

## 4. Results

In this section we study how a signal injected in population 1 or 2 is transmitted to the rest of the populations in the circuit. We mainly analyze the transmission when there is a frequency detuning between the oscillation frequencies of the populations and the transmission time of the signal between populations is non-zero. We consider two particular circuits: the V-motif and the C-motif (see Figure 1).

### 4.1. Synchronization and Phase-Locking in the V-Motif Circuit

**Figure 2:**
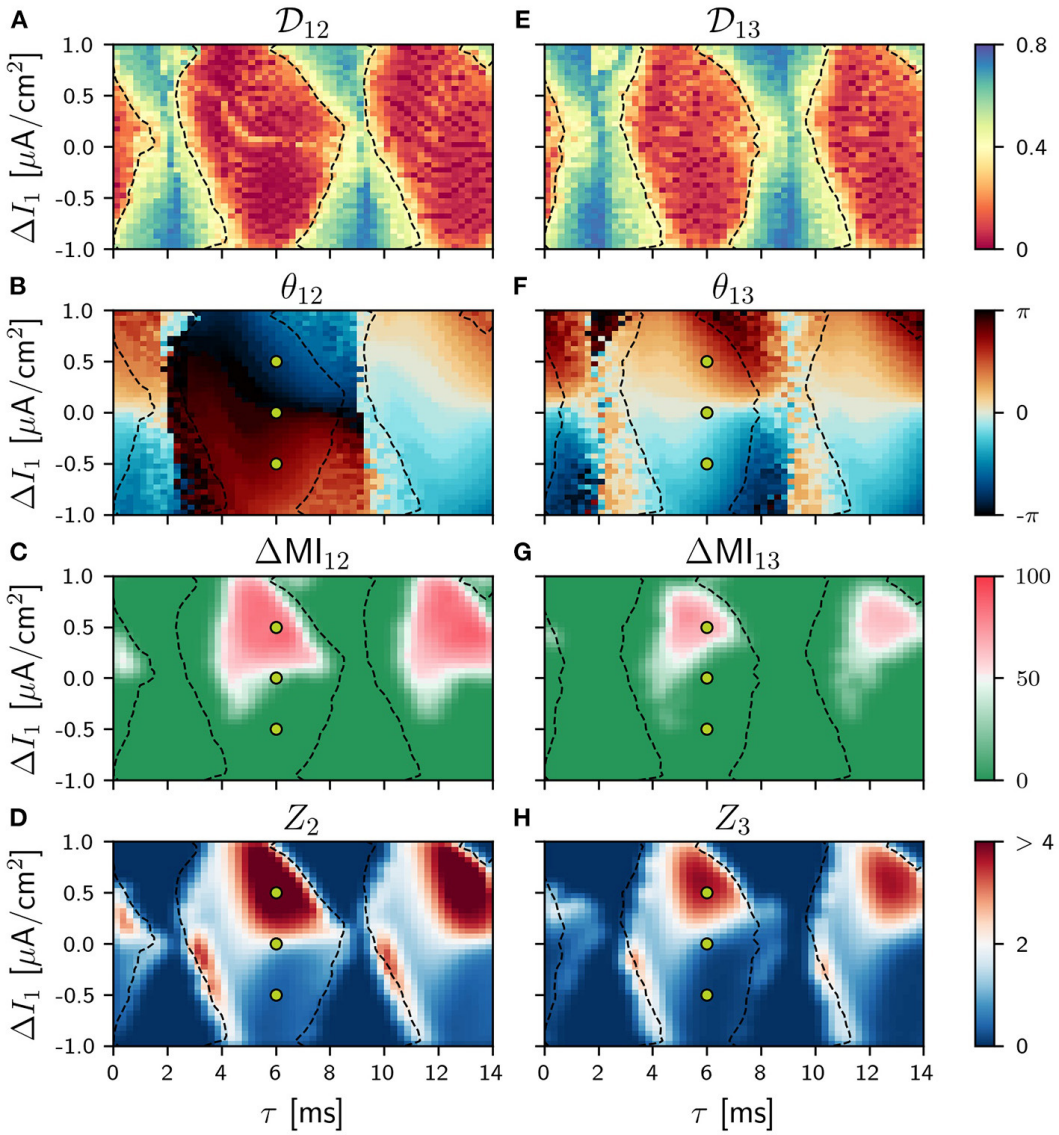
Population 1 as the sender in the V-motif. Phase locking index $D_{ij}$ **(A,E)**. Phase difference θ_*ij*_ **(B,F)**. Difference ΔMI_*ij*_ **(C,G)** when a slow modulation is injected. Integral of the absolute value of the nPRC *Z*_*i*_ **(D,H)** when a fast signal is injected. All of them as a function of the delay and the frequency mismatch Δ*I* in population 1.

We start showing our results for the case of the V-motif, i.e., when the cortico-cortical connections were neglected. (*g*_13_ = *g*_31_ = 0 μS/cm^2^). We assume here that the coupling strengths between populations are symmetric, i.e. *g*_12_ = *g*_21_ =*g*_23_ = *g*_32_ (results for asymmetric cases are shown in the Supplementary Material). As we mentioned before (see Methods), each isolated neural population exhibits a high synchronized behavior which is also kept when they are coupled (see Pariz et al., 2018). This fact allows us to determine the phase difference between the oscillations of the populations. Since we are interested in studying the information transmission from one outer population or from the relay population to the others, we must consider two situations for the analysis of the locking regions: when the frequency mismatch is applied to population 1 (or equivalently to population 3) and when it is applied to population 2. When analyzing the phase-locking regions, estimated by computing the phase-locking index (see Methods), with respect to our control parameters (τ and Δ*I*), three regions are generally found delimited by two non-locked regions (see Figures 2A,E and Supplementary Figures 2–5). The shape of the non-locked regions (and consequently of the phase-locking regions) changes as a function of the synaptic weight *g* and the induced frequency mismatch. However, the emergence of these regions is a general property of delay-coupled dynamical systems (Mirasso et al., 2017).

**Figure 3:**
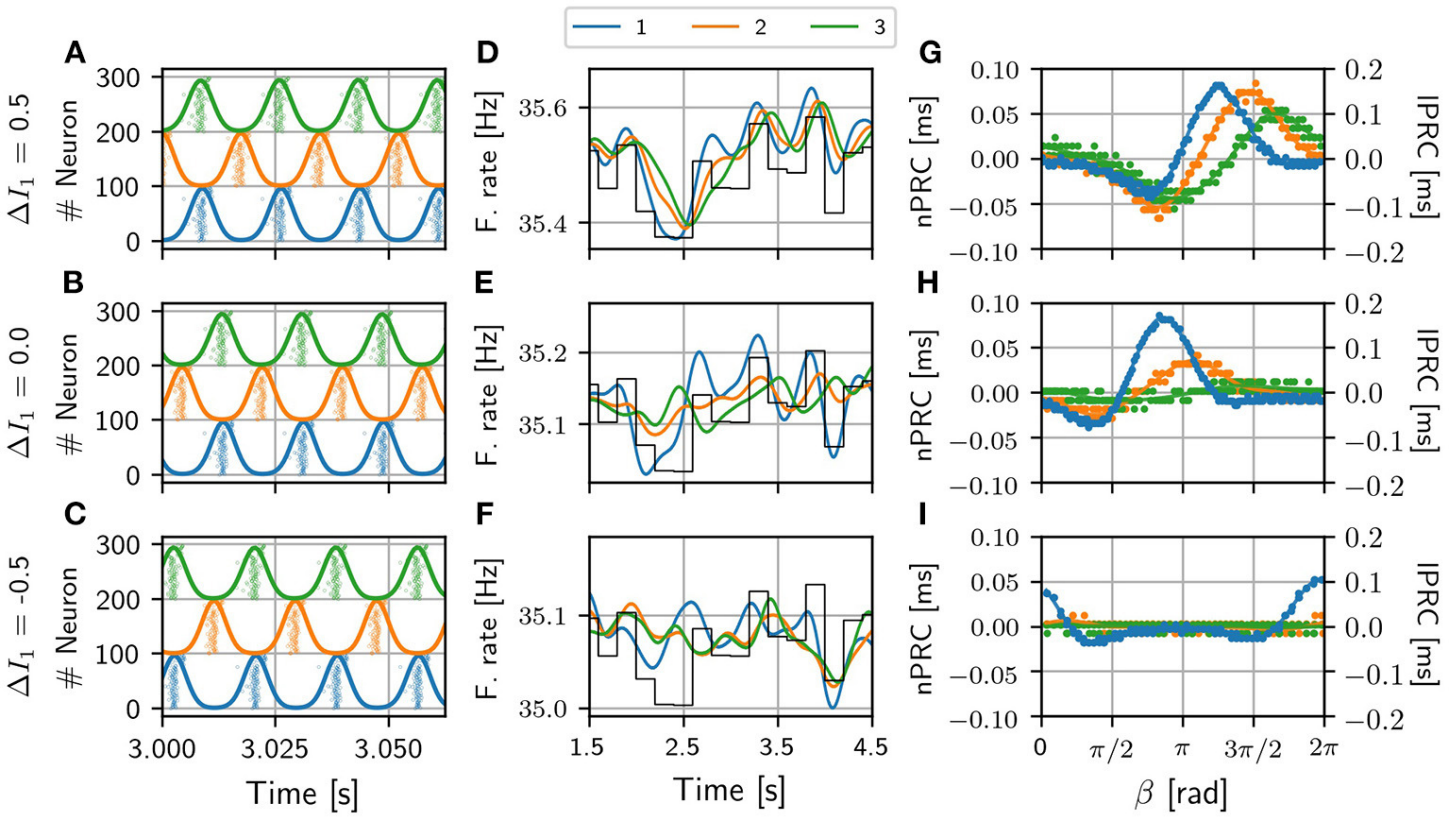
V-motif. Examples of the activity of the three populations and their corresponding firing rate for different values of Δ*I* in the absence of an external signal **(A–C)**. Examples of firing rates of the three populations for different values of the frequency mismatch Δ*I* when a slow arbitrary signal (black line, arbitrary units) is applied to population 1 **(D–F)**. Examples of local and non-local phase response curves of the sender and receiving populations, respectively, for the same different values of frequency mismatch Δ*I* when a fast pulsating signal is applied in population 1 **(G–I)**. Note that the scale of lPRC and nPRC are different for a better visualization. Delay τ = 6 ms in all cases.

**Figure 4:**
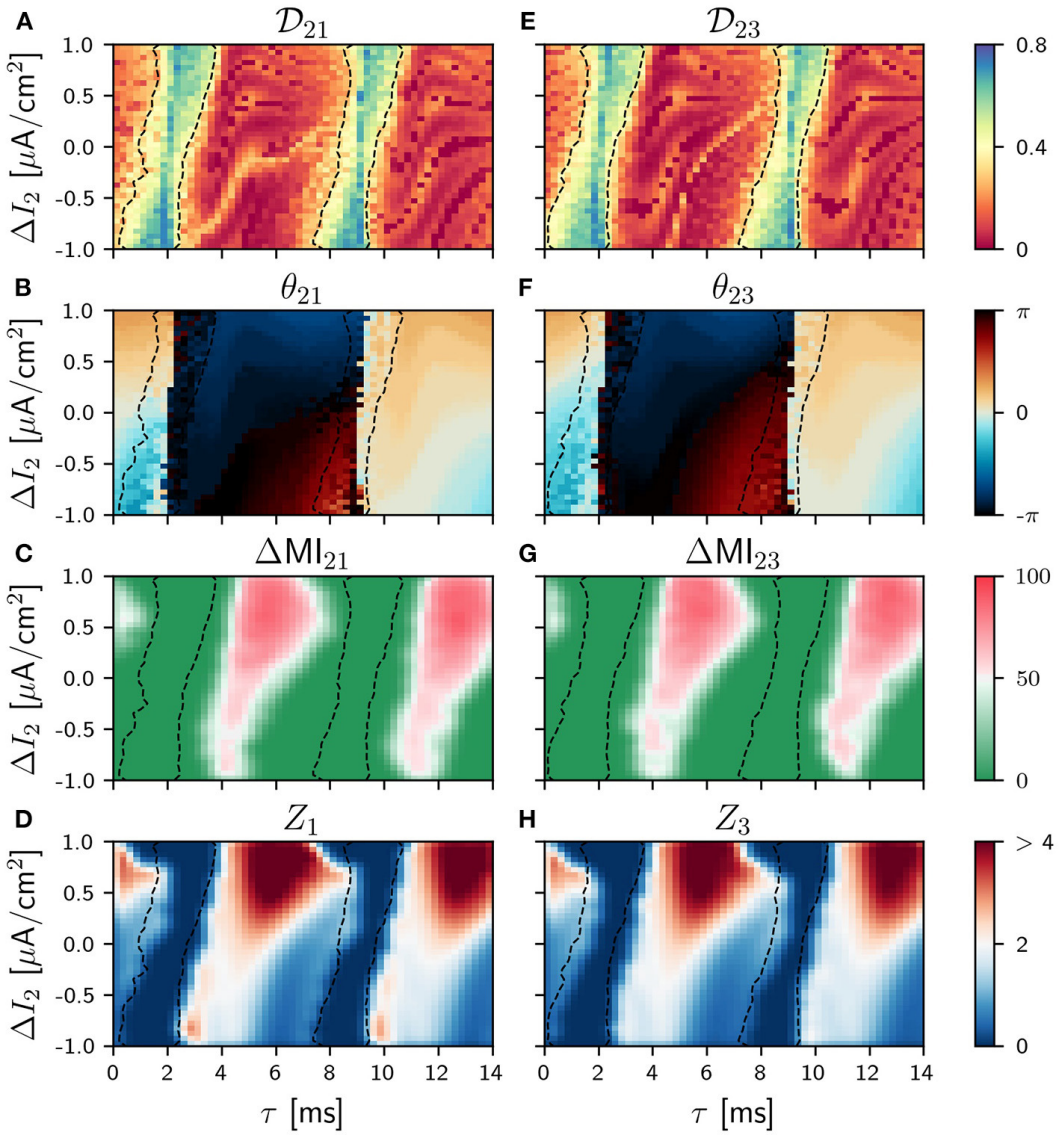
Population 2 as the sender in the V-motif. Phase locking index $D_{ij}$ **(A,E)**. Phase difference θ_*ij*_ **(B,F)**. Difference ΔMI_*ij*_ **(C,G)** when a slow modulation is injected. Integral of the absolute value of the nPRC *Z*_*i*_ **(D,H)** when a fast signal is injected. All of them as a function of the delay and the frequency mismatch Δ*I* in population 2.

The locking regions between populations 1 and 2 (1 and 3) are shown in the Δ*I* vs. τ phase space in of Figure 2E when the detuning Δ*I* is applied in the population 1 and in Figure 4 when applied in population 2. As expected, these locking regions are slightly larger between populations 1 and 2 than between populations 1 and 3 although a high value for the phase index is found in both cases. The phase at which the oscillations of populations 1 and 2 (1 and 3) lock are shown in Figures 2B, 4F. In the absence of frequency mismatch Δ*I* = 0, we observe the expected zero-lag synchronization between population 1 and 3. Also for small and high (close to the oscillation period) values of the delay, populations 1 and 2 are almost in-phase. However, for intermediate values they exhibit an anti-phase dynamics, as previously observed (Vicente et al., 2008; Esfahani and Valizadeh, 2014; Mirasso et al., 2017). Examples of the activity of the three populations and their firing rates can be seen in Figures 3A–C for differents values of the detuning when the connection delay is 6 ms. For Δ*I* ≠ 0 and for small or large delays, the locking phase is no longer zero but it is positive (delayed synchronization) for positive detuning and negative (anticipated synchronization) for negative detuning (Mirasso et al., 2017). It is worth mentioning that analysis considering different values of the coupling strength were undertaken obtaining qualitatively similar results to those shown in Figure 2 (see Supplementary Material).

### 4.2. Information Transmission in the V-Motif Circuit

Depending on whether we analyze the transmission of a slow (modulation) or fast (chain of pulses) signal, we use different tools. The effect of the slow signal is translated into a modulation of the firing rate, keeping oscillation frequency of the population constant (in those cases where phase locking occurs), while each pulse of the fast signal modifies the period of an oscillation cycle. Therefore, these two effects occur at different time scales that require different tools to quantify them.

For the first case, as explained in the material and methods section, we used two different measurements: the zero-lag cross-covariance (ZLC) between the firing rate of the receiving population and the injected signal, and the difference ΔMI_*ij*_ computed from the time-delayed mutual information between the firing rates of the sender and receiver populations.

We show in Figure 2 panels C and G, the results for the ΔMI_*ij*_ when the population 1 (cortical area) acts as the sender and a slow modulation signal is injected (locking areas from panel A and E are also included here with dashes lines). Also in the Supplementary Figure 7 we have shown the results for asymmetric cases. The corresponding ZLC results are shown in Supplementary Figure 6.

Generally, regions with high values of ΔMI_*ij*_ in Figure 2 and with high values of ZLC in Supplementary Figure 6 qualitatively match despite the fact that they quantify different properties. The covariance determines the similarity of the activity between two populations, while the difference ΔMI_*ij*_ indicates directionality and strength of the transmission. However, we checked that these two quantities are more correlated the closer the phase-locking index tends to zero, i.e., perfect locking (see Supplementary Figures 12–14). Moreover, we found that in the cases where the values of ΔMI_*ij*_ and cross-covariance are the highest, the phase-locking index is very close or to zero, or equivalently, there is a constant well-defined phase relation. This result is in a good agreement with the Communication Through Coherence hypothesis (Fries, 2015), where a well-defined phase relation is required to enhance the communication between neural populations.

As it can be observed, the optimal way to transmit a slow modulation signal from 1 to 2 and then to 3 is by imposing a positive frequency mismatch (positive Δ*I*; higher oscillation frequency) in the sender population since for negative values of the frequency mismatch the transmission is very poor. These findings are in a good agreement with previous results (Pariz et al., 2018, 2021). Yellow points in Figures 2C,G refer to the selected values of the detuning Δ*I* which characterize the temporal series shown in Figures 3D–F for a delay τ = 6 ms. For those points that lie within the red region in Figure 3, the firing rate of the receiver populations follows quite well the injected signal, while those outside the red regions do not. The fact that the ΔMI_1, 2_ is high reflects the similarity between the slow variations in the firing rates of the two populations, at a time scale which is much longer than that of the gamma oscillations. Interestingly, for some cases of negative detuning the time evolution of the firing rates are quite similar to each other, however, none of them follow the injected signal (not shown).

Similar results were obtained when we injected a fast pulsating signal instead of the slow signal. For the fast signal injection, we computed the integral of the absolute value of the non-local phase response curve (nPRC) of the receiving populations (see Methods). The results are shown in Figures 2D,H where it can be observed that the regions where a better transmission occurs match quite well with those of the slow modulation case. For these colormaps, we impose *Z*_*i*_ = 0 when the resultant dynamics does not exhibit a PRC shape-like, assuming that there is no signal transmission. We also show some examples in Figures 3G–I of the lPRC of the sender population (blue) and the nPRCs of the receiving populations (orange and green) for the values of the parameters indicated in yellow in Figures 2G,H. For the points laying outside the locking areas, an analytical expression for the fitting cannot be obtained. In general we observed the information transmission, either rate or spike coding, changes with the strength *g*, improving as *g* increases (see Supplementary Figure 8).

The situation drastically changes when the sender is population 2 (the thalamus in our cortico-thalamic-cortical assumption) and the system maintains symmetry even when a frequency mismatch is applied.

The information transmission of a slow signal to both populations 1 and 3 are equivalent, as it can be seen in Figures 4C,G (see Supplementary Figure 10 for asymmetric cases and Supplementary Figure 9 for ZLC). Furthermore, the transmission is possible in this case even for negative values of the frequency mismatch for intermediate and long delays. This result is equivalent to the case of two mutually-coupled neural population reported in Pariz et al. (2021) and is a direct consequence of the symmetry of the system. However, we found that the capability of the signal transmission over this condition is reduced, as expected, as the cortico-thalamic synaptic strength increases (results not shown).

A similar behavior is observed when a fast arbitrary signal is injected, as it can be seen in Figures 4D,H where the patterns of *Z*_*i*_ are shown (see Supplementary Figure 6 for asymmetric cases).

### 4.3. Synchronization and Phase-Locking in the Circular Motif Circuit

**Figure 5:**
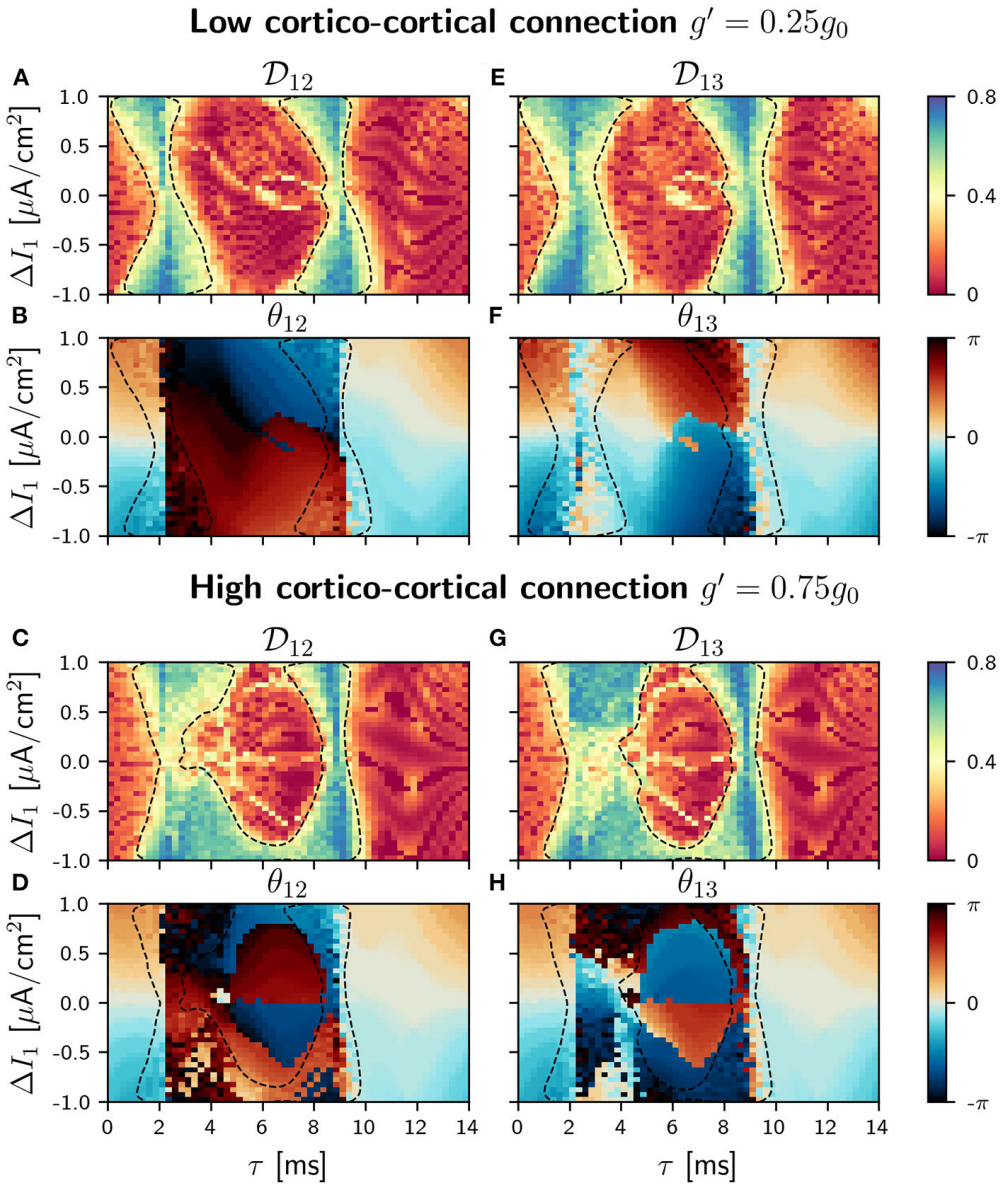
C-motif: Phase locking $D_{ij}$ for *g*′ =0.25*g*_0_ **(A,E)** and *g*′ =0.75*g*_0_ **(C,G)**. Phase difference θ_*ij*_ for *g*′ =0.25*g*_0_ **(B,F)** and *g*′ =0.75*g*_0_ **(D,H)**. All of them as a function of the delay τ and the frequency mismatch induced by a change Δ*I* in population 1.

**Figure 6:**
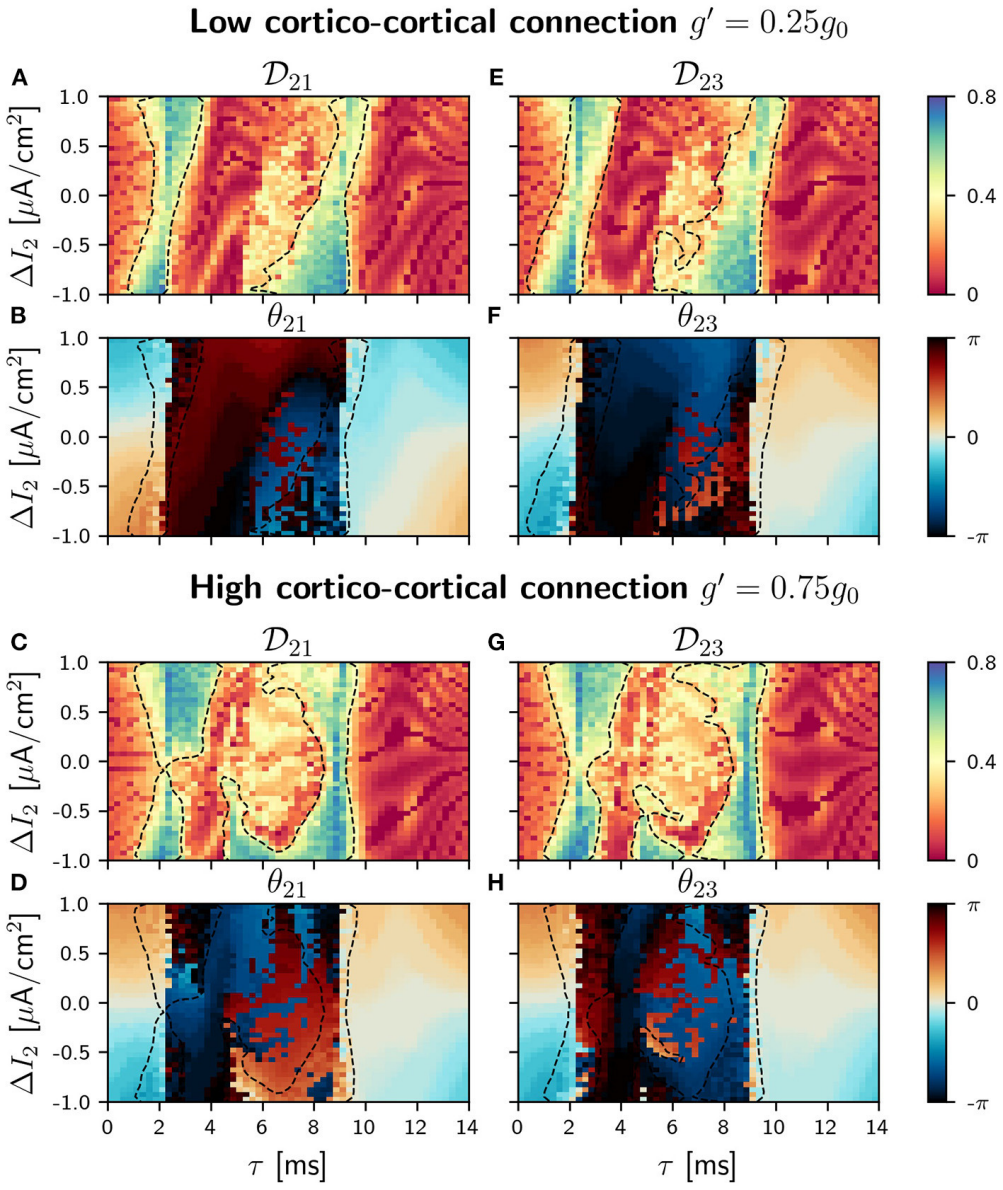
C-motif: Phase locking $D_{ij}$ for *g*′ =0.25*g*_0_ **(A and E)** and *g*′ =0.75*g*_0_ **(C and G)**. Phase difference θ_*ij*_ for *g*′ =0.25*g*_0_ **(B and F)** and *g*′ =0.75*g*_0_ **(D and H)**. All of them as a function of the delay τ and the frequency mismatch induced by a change Δ*I* in population 2.

With the addition of a cortico-cortical connection, the circuit has now a ring topology. We considered four different values of the synaptic strength *g*′ between the outer populations (= *g*_13_ = *g*_31_): 0.05*g*_0_, 0.25*g*_0_, 0.50*g*_0_, 0.75*g*_0_, always weaker than the cortico-thalamic and the thalamo-cortical connections. The results presented in this section account only for two of these values, *g*=0.25 *g*_0_ (low) and *g*=0.75 *g*_0_ (high). Results for the other *g*'s values are included in the Supplementary Material. The strength of the thalamo-cortical and the cortico-thalamic synapses is kept constant and equal to *g*_0_. We proceed in the same way as before, analyzing first the phase differences distributions and the conditions of phase-locking in the parameter space. We show in Figures 5, 6 the phase-locking index and the phase differences for low and high values of cortico-cortical synaptic conductance *g*′ when the detuning is applied in population 1 and 2, respectively (see Supplementary Figures 15–18 for all the different values of *g*′).

According to the coupled oscillators theory, in the absence of frequency mismatch, two identical and mutually coupled oscillators with delay in their connections can only be in two regimes of synchronization: in-phase and anti-phase (DHuys et al., 2008). Consequently, the addition of the third connection to our V-motif rises the competition between the V-motif dynamics and the dynamics preferred by the mutual coupling of the external populations. While the V-motif structure tends to synchronize populations 1 and 3 at zero-phase (or close to zero-phase) independently of the connection delay, the additional direct connection between populations 1 and 3 tends to synchronize them in anti-phase at intermediate delay values.

In our circular motif and for small values of the synaptic strength *g*′ the effect of the new connections is very small, as it can be seen in Figures 5A,B,E,F,H. However, as soon as we increase *g*′, its effect becomes noticeable in the phase differences and in the locking regions that reduced their size, as it can be seen in Figures 5C,D,G,H. The competition between the zero (or almost zero) phase locking of the V motif and the π (or almost π) phase locking of the mutually coupled populations only occurs in the interval of intermediate values of the delay, since for small and large delay values both systems exhibit in-phase dynamics. We observed, in this delay window, that the phase-locking index increased as the synaptic strength *g*′ was increased (results not shown).

### 4.4. Information Transmission in the Circular Motif Circuit

**Figure 7:**
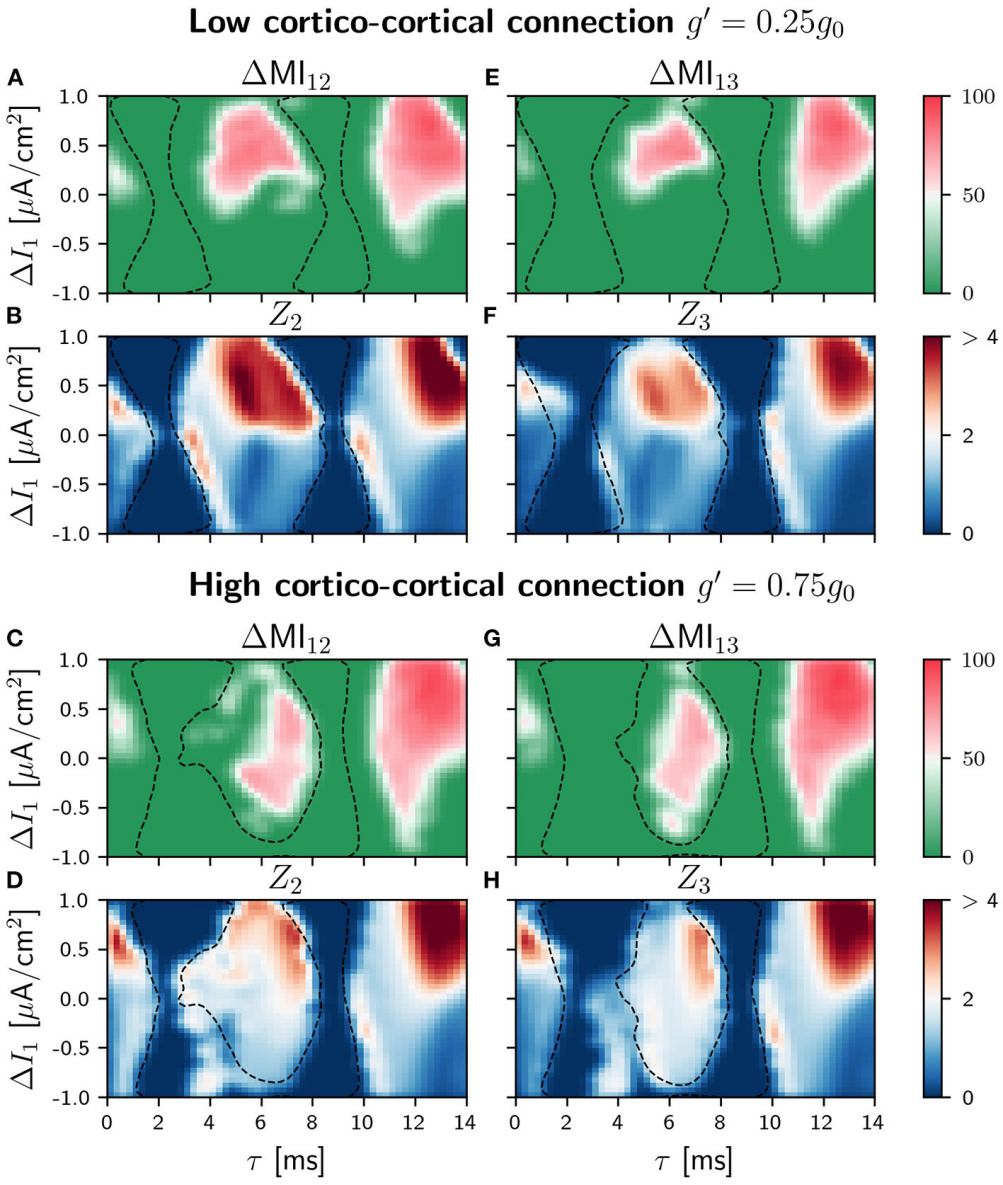
Population 1 as the sender in C-motif: Difference ΔMI_*ij*_ for *g*′ =0.25*g*_0_ **(A and E)** and *g*′ =0.75*g*_0_ **(C and G)** when a slow modulation is injected. Integral of the absolute value of the nPRC *Z*_*i*_ for *g*′ =0.25*g*_0_ **(B and F)** and *g*′ =0.75*g*_0_ **(D and H)** when a fast signal is injected. All of them as a function of the delay τ and the frequency mismatch induced by a change Δ*I* in population 1.

As a consequence of the mentioned competition, the efficiency of the information transmission in the circuit is expected to be affected. Again, we explored the propagation of slow and fast signals when the sender was the population 1 or the population 2. The differences ΔMI_*ij*_ when the slow modulation was injected in population 1 are shown in Figures 7A,E for small *g*′ and Figures 7C,G for large *g*′ (see Supplementary Figure 20 for all the values considered for *g*′ and Supplementary Figure 19 for the correspondant ZLC results). As it can be seen, the effect of the cortico-cortical connection is noticeable for $g^{\prime }=0.75g_{0}$. Comparing with the case in which *g*′ = 0.25*g*_0_, the shapes of the regions where the transmission is good for the intermediate delay values have changed, and the values of ΔMI_*ij*_ (strength and directionality of the transmission) become smaller. However, the situation is very different with respect to the case of the V-motif, since now the transmission is enhanced for negative detuning (negative values of the current mismatch Δ*I*) both for populations 1 or 2 and 1 and 3. Furthermore, this effect is also noticeable for high values of delay. We observed in this case that the communication for negative detunings improves as the synaptic weight *g*′ is increased (results not shown).

When we considered the injection of a fast arbitrary signal, the situation was similar to those of the case of slow signals as it can be seen in Figures 7B,D,F,H for low an high values of the conductance *g*′, respectively. For *g*′ = 0.75 and intermediate values of the delay, the transmission is moderately enhanced for negative values of the detuning in contrast to the V-motif case. Furthermore, for high values of the delay, the transmission in general enhanced but without an important effect for negative values of the frequency mismatch (see Supplementary Figure 21 for different values of *g*′).

**Figure 8:**
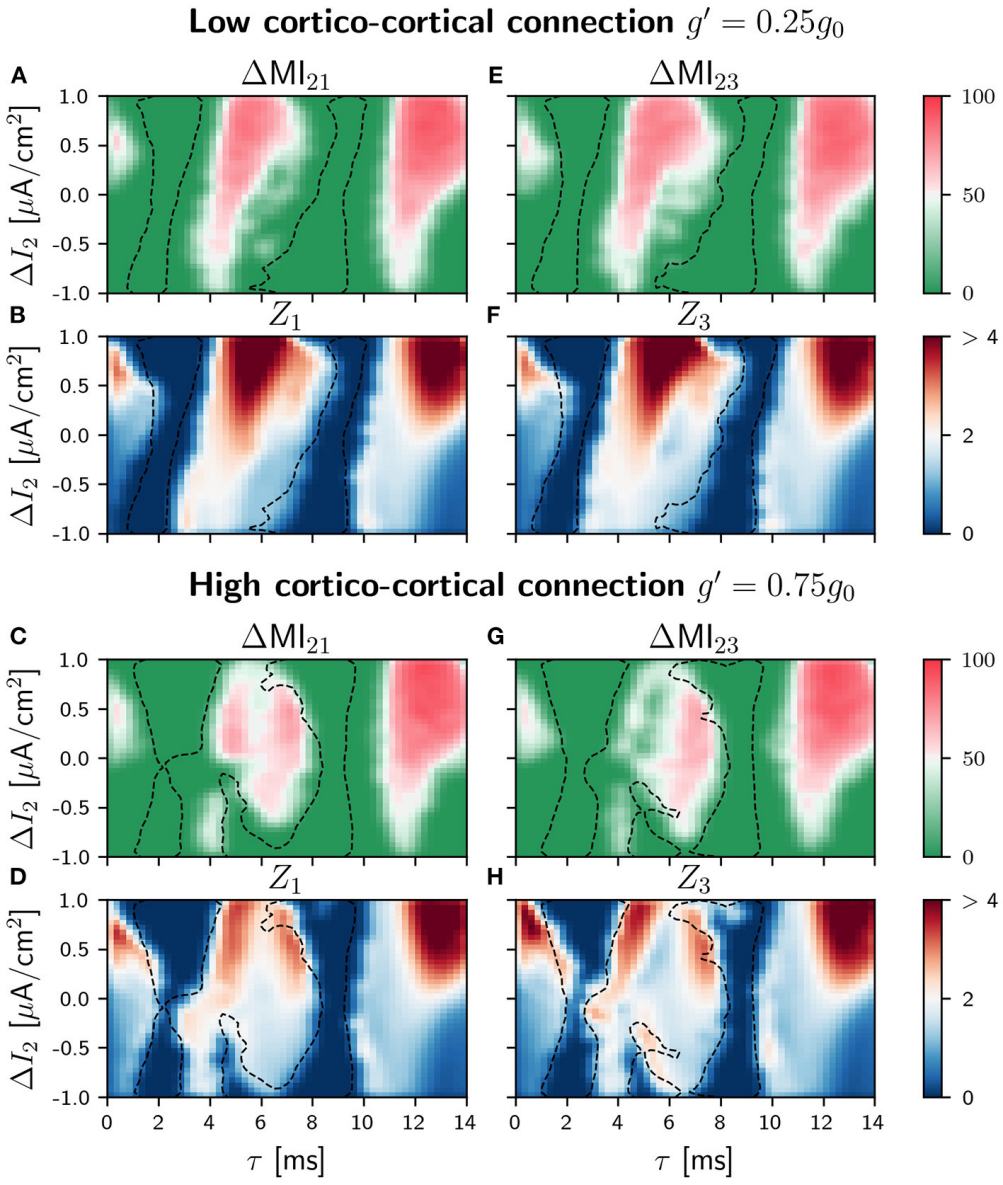
Population 2 as the sender in C-motif: Difference ΔMI_*ij*_ for *g*′ =0.25*g*_0_ **(A,E)** and *g*′ =0.75*g*_0_ **(C,G)** when a slow modulation is injected. Integral of the absolute value of the nPRC *Z*_*i*_ for *g*′ =0.25*g*_0_ **(B,F)** and *g*′ =0.75*g*_0_ **(D,H)** when a fast signal is injected. All of them as a function of the delay τ and the frequency mismatch induced by a change Δ*I* in population 2.

As for the case of slow signal modulation, important changes in the behavior of the system occur in the intermediate region when the sender is the population 2, as it can be seen in Figure 8 (see Supplementary Figure 21 for the different values of *g*′). Again the regions of signal transmission depend less on the detuning and the delay.

In general, our results indicate that the quality of the signal transmission does not depend much on whether the external modulation is slow or fast, neither for the V-motif nor for the circular motif. What significantly affects transmission is the addition of the cortico-cortical link between populations 1 and 3. In this case, transmission becomes, unexpectedly, less robust and effective, but allowing information transmission also for negative detunings.

## 5. Discussion

In this paper we have studied transmission of signals in neuronal circuits considering two different canonical motifs: the V-motif that consists of a chain of three bidirectionally coupled neural populations and the circular motif, which results from adding bidirectional connection between the populations at the end of the chain. These simple neural motifs are inspired in the cortico-thalamo-cortical network and allow us to address open questions about the condition for efficient information transmission in this circuit. We identify two operation modes that can be dynamically switched by modulating the strength of the direct cortico-cortical connection. When this connection is weak, the V-motif dynamics predominate supporting robust transmission from the thalamus to the cortex as well as in feedback cortico-thalamic direction. When the strength of cortico-cortical connection increases, C-motif dynamics facilitate the coexistence of the above canonical thalamo-cortical transmission with a cortico-thalamo-cortical association loop. Thus, direct cortico-cortical and indirect transthalamic communication cooperate in cortical computations. We hypothesize that the first operation mode supports unimodal sensory transmission and the second multimodal integration and feature binding.

To investigate information transmission in the above system, we systematically explored the consequences of varying two important parameters, detuning or frequency mismatch between the populations (produced through a change in the bias current Δ*I* applied to one population, the sender), and the delay (τ) in the connections between populations. The first would be dynamically controlled by modulating the afferent synaptic strengths, neuronal excitability or the excitation/inhibition balance in the networks, and the second is hardwired in the system and depends on the axonal length and conduction velocities. From previous works, it is known that applying a positive detuning (resulting in a higher oscillation frequency) to the sender population, enhances the communication in the network (Sancristóbal et al., 2014; Kirst et al., 2016; Palmigiano et al., 2017; Pariz et al., 2018, 2021). However, this is true when we consider small values of the delay τ. For larger delay values, it is the combination of the detuning and the delay that determine the efficacy and the preferred direction of the signal transmission (Pariz et al., 2021).

Based on the theory provided by Pariz et al. (2021), phase relations between pair of the bidirectionally coupled oscillators can determine the amount of signal/information transfered between them, once their PRCs are known. For symmetric bidirectionally coupled oscillators both 0 and π phase difference can be stable depending on the delay time and their PRCs. In the three network motifs, the organization of the connections can also affect the phase relation. In the absence of the cortico-cortical connections, and depending on the delay, each pair of adjacent nodes can take 0 or π phase, and in either case the outer populations favor the zero phase lag (Gollo et al., 2010, 2014; Mirasso et al., 2017). In a symmetric circular motif, a new state emerges where the three populations tend to lock at 2π/3 phase difference compatible with the symmetry of the motif. For small values of the cortico-cortical connection the former states have larger basin of attraction while this latter state only appears for large enough values of the cortico-cortical connection strength. In this case, the pattern of information transfer deviates from that of the relay motif.

As it is shown in Pariz et al. (2021), the amount of signal transmission between the neuronal populations depends on the slope of the phase response curve of the receiver population (nPRC) at the time that it receives the spiking activity of the sender population. In this way not only the phase relation between the two populations and the delay time, but also the PRC of the populations are important in the pattern of information transmission between neuronal populations. Population PRCs are shown to depend on the mechanism of the generation of the oscillations in the excitatory-inhibitory networks (Dumont and Gutkin, 2019) and consequently, the internal dynamical properties of the EI networks also affect the information transfer in brain circuits. Moreover, the preference of the system for a positive detuning as compared to the negative one is due to the asymmetric shape of phase response curve of the realistic neurons (Sadeghi and Valizadeh, 2014) and neuronal populations (Dumont and Gutkin, 2019), as is shown in Pariz et al. (2021). The same results seen for the three neurons motifs in this study, confirm that the shape of the PRC and in particular its symmetry properties can fundamentally affect information transfer in the brain.

In agreement with previous findings, our results show that in general positive detuning enhances transmission between populations. However, there are cases in which a good transmission is feasible even for negative detuning values. In the V-motif, this happens when the intermediate population (the thalamic relay) acts as the sender population. When the signal is injected in one of the external populations (cortical), good transmission always requires positive values of the detuning, that is, higher frequencies in the cortical vs. the thalamic population. Following the biological analogy, our results suggest that the canonical thalamo-cortical sensory transmission would be preserved for a broad range of thalamic frequencies (positive and negative detuning), thus facilitating the coexistence of top-down and bottom-up computations required for proper sensory integration (Hirsch et al., 2015). Cortico-thalamic transmission (top-down) in the V-motif would be restricted to positive detuning (when cortical frequency is higher than that of the thalamus). This operation mode is compatible with the canonical view of the thalamic function (Sherman, 2016).

Interestingly, different operation modes appear by adding a direct link between the outer populations (cortico-cortical connection generating the C-motif). In this condition, higher cortico-cortical synaptic weights (*g*′) facilitate effective communication in the indirect cortico-thalamo-cortical pathway for negative detuning values, a communication channel that did not exist in the V-motif. Accordingly, for small values of cortico-cortical connection, the dynamics is mainly governed by that of the V-motif. Therefore, the strength of the cortico-cortical connection may control switching between distinct dynamic modes.

Quantitatively, the V-motif (or the C-motif with low cortico-cortical connection strength) is a better configuration for efficient signal transmission but, on the other hand, the circular motif with higher cortico-cortical connectivity allows new channels of communication, although with lower overall efficiency. The later generates cortical associative loops in which both, the direct cortico-cortical and the indirect transthalamic pathways, play distinct but fundamental roles. We hypothesize that the first operation mode supports the canonical function of the thalamus in sensory transmission and the second would be ideally suited for the integration of cortically segregated computations and feature binding (Singer, 2007; Uhlhaas et al., 2009; Gollo et al., 2010).

Our study has some limitations. All these results have been obtained in a highly coherent and synchronized regime. The intrinsic dynamics of each population exhibited a single oscillation frequency which cannot fully represent the realistic dynamics of cortical networks. It is well known that brain functions are associated with different frequency bands, and synchronization occurs in short periods from a weakly coherent scenario (Xing et al., 2012). Previous studies have proposed neural network models to reproduce this complex dynamics (Tort et al., 2013; Palmigiano et al., 2017) and others suggested the important role of slow synapses (Cannon et al., 2014), not considered in our model. Therefore, further work must be done considering richer neural dynamics which will help to further explain the underlying circuit mechanisms of information transmission between brain areas.

In conclusion, signal propagation in V- and C-motifs resembling those found in the thalamo-cortical system was systematically studied. Complex but distinct operation modes emerge in this relatively simple system with three neuronal populations and two main variables, connection delay and frequency detuning. An important modulatory role was identified for the cortico-cortical connection strength in the selection between operation modes. In a neurobiological thalamo-cortical context, modes switching would flexibly support the alternation between computations with a predominant sensory processing function, with robust thalamo-cortical transmission and top-down cortico-thalamic modulation (i.e., attentional modulation), with feature binding, and cortical integrative computation.

## Data Availability Statement

The raw data supporting the conclusions of this article will be made available by the authors under request, without undue reservation.

## Author Contributions

AV, SC, and CM contributed to conception and design of the study. JS-C and AP performed simulations and prepared the figures. All authors contributed to manuscript revision, read, and approved the submitted version.

## Conflict of Interest

The authors declare that the research was conducted in the absence of any commercial or financial relationships that could be construed as a potential conflict of interest.

## Publisher's Note

All claims expressed in this article are solely those of the authors and do not necessarily represent those of their affiliated organizations, or those of the publisher, the editors and the reviewers. Any product that may be evaluated in this article, or claim that may be made by its manufacturer, is not guaranteed or endorsed by the publisher.
