# Supplementary material for: Information Transmission in Delay-Coupled Neuronal Circuits in the Presence of a Relay Population
Source: Front Syst Neurosci. 2021 Jul 29;15:705371. doi: 10.3389/fnsys.2021.705371 (PMC8357994; doi:10.3389/fnsys.2021.705371)
Supplement: Supplementary file 1 [file Data_Sheet_1.PDF]

# Supplementary Material

## 1 POPULATION DYNAMICS

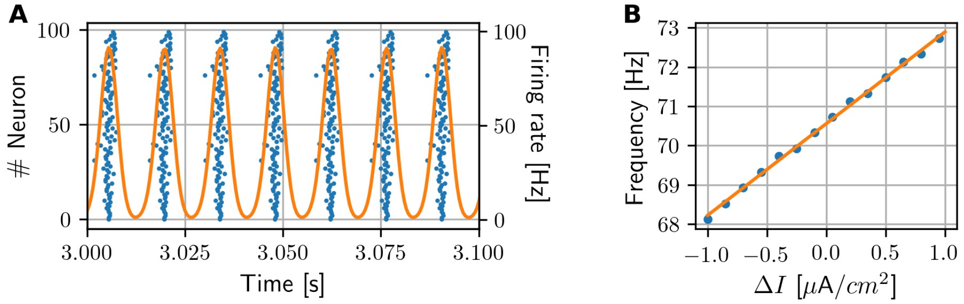


**Figure S1.** Raster plot and firing rate of one isolated neural population with $I = I_0 = 11\ \mu\text{A}/cm^2$ (A). Linear dependency of the oscillating population frequency on the detuning $\Delta I = I - I_0$ (B).

## 2 V-MOTIF: SYNCHRONIZATION AND PHASE-LOCKING

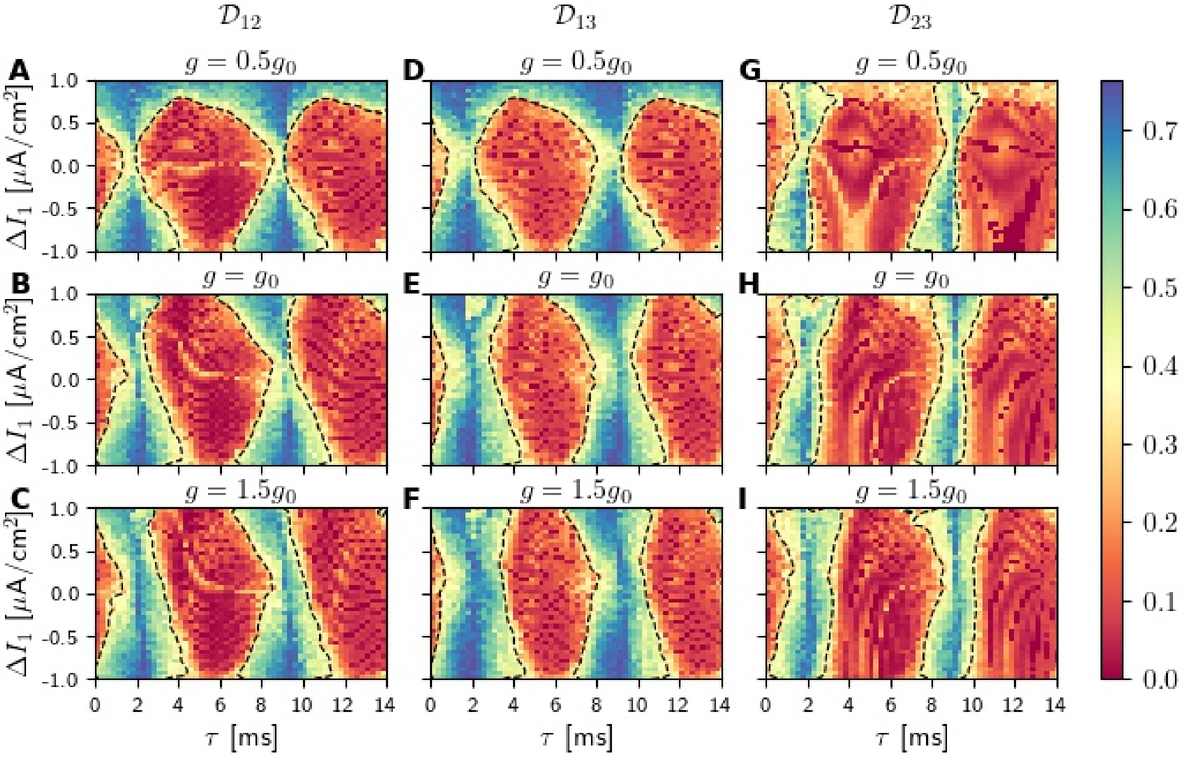


**Figure S2. V-motif**: Phase locking between population 1 and 2 (A-C), population 1 and 3 (D-F), and population 2 and 3 (G-I) as a function of the delay $\tau$ and the frequency mismatch $\Delta I$ in population 1 for different values of the synaptic strength $g$. The phase locking is measured with the index $\mathcal{D}_{ij}$ which implies a better locking as its value is closer to zero (see Methods in the main text).

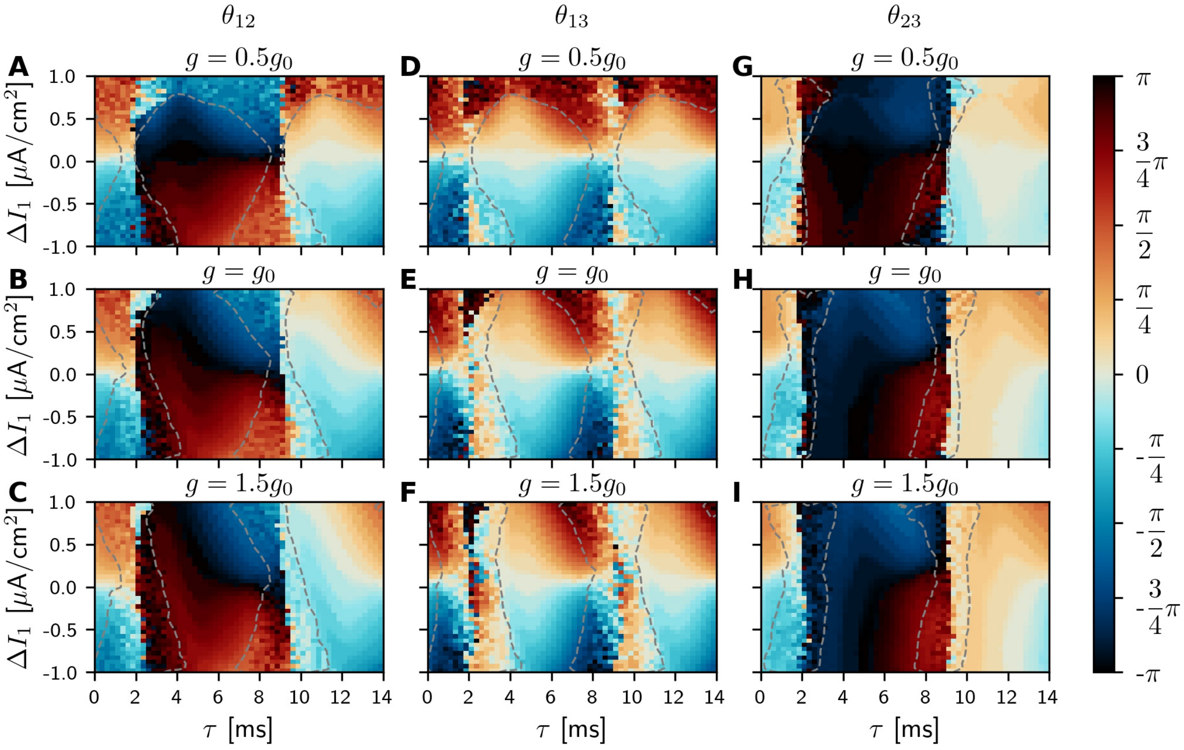


**Figure S3. V-motif**: Phase difference between population 1 and 2 $\theta_{12}$ (A-C), population 1 and 3 $\theta_{13}$ (D-F), and population 2 and 3 $\theta_{23}$ (G-I) as a function of the delay $\tau$ and the frequency mismatch $\Delta I$ in population 1 for different values of the synaptic strength $g$.

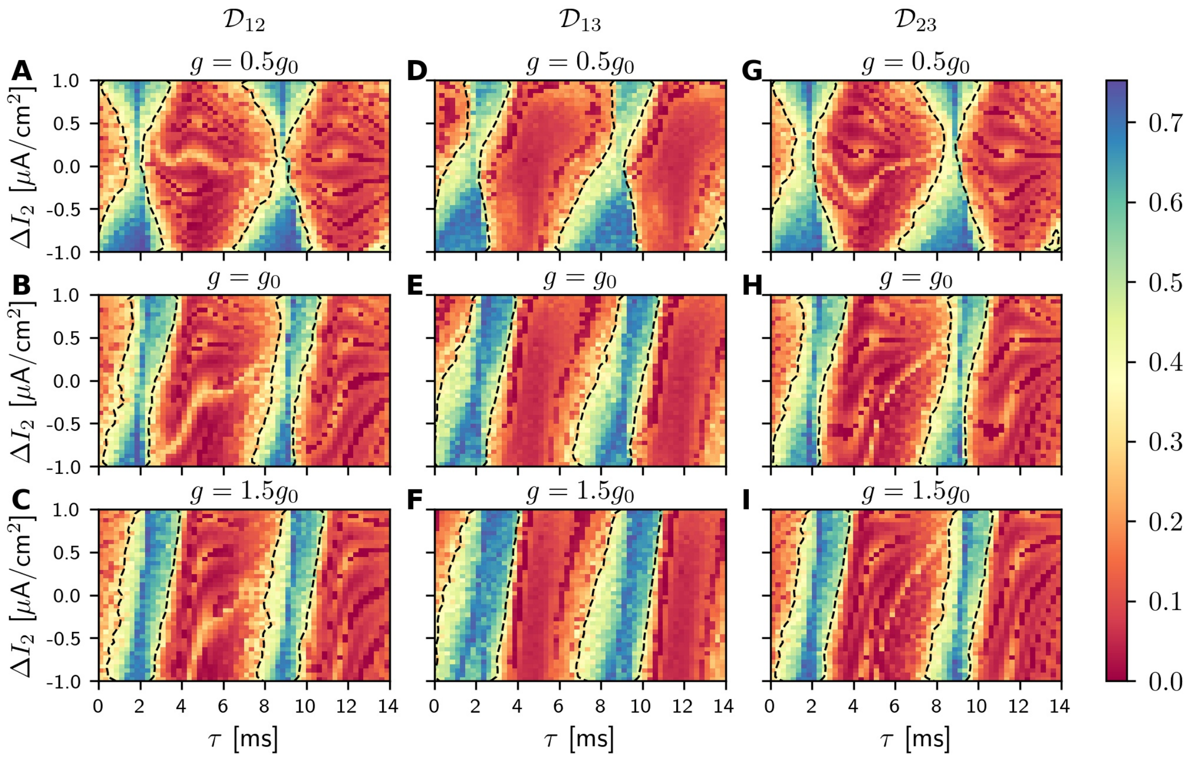


**Figure S4. V-motif**: Phase locking between population 1 and 2 (A-C), population 1 and 3 (D-F), and population 2 and 3 (G-I) as a function of the delay $\tau$ and the frequency mismatch $\Delta I$ in population 2 for different values of the synaptic strength $g$.

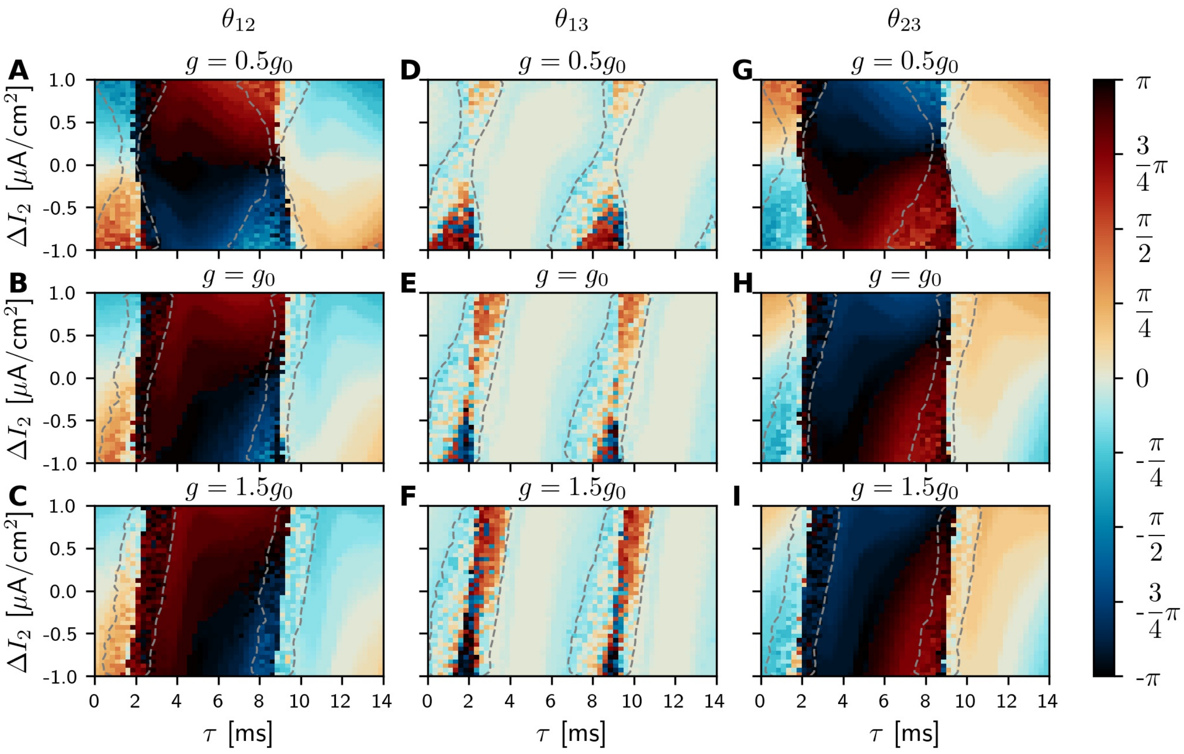


**Figure S5. V-motif**: Phase difference between population 1 and 2 $\theta_{12}$ (A-C), population 1 and 3 $\theta_{13}$ (D-F), and population 2 and 3 $\theta_{23}$ (G-I) as a function of the delay $\tau$ and the frequency mismatch $\Delta I$ in population 2 for different values of the synaptic strength $g$.

## 3 V-MOTIF: INFORMATION TRANSMISSION

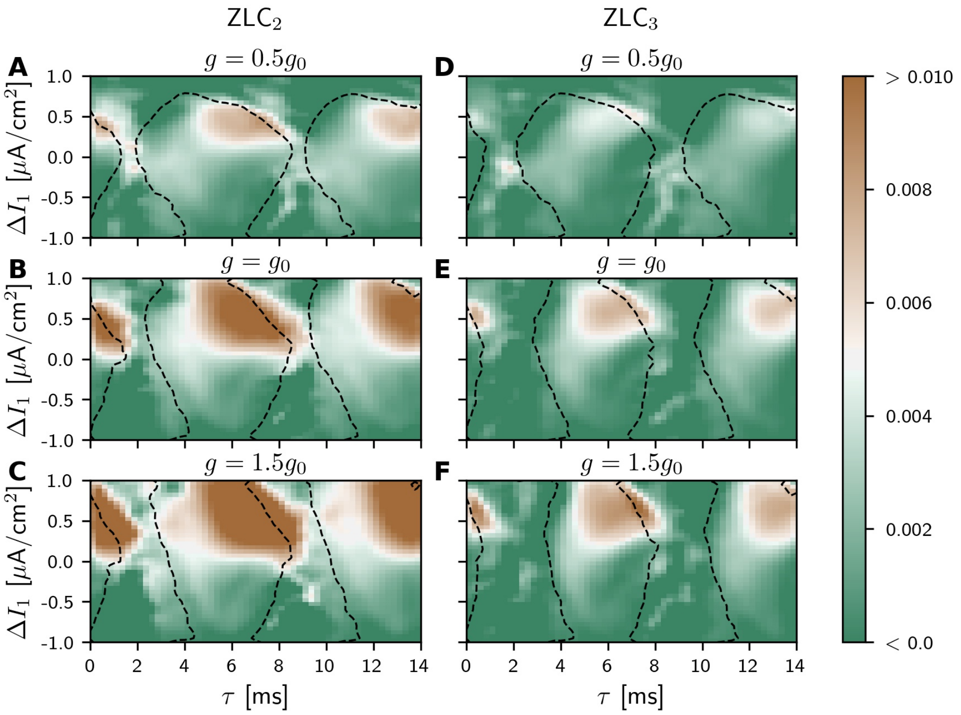


**Figure S6. V-motif**: Zero-lag cross covariance (ZLC) of the firing rates of the second (A-C) and third (D-F) population with the slow modulation injected as function of the delay $\tau$ and the frequency mismatch $\Delta I$ in population 1 for different values of the synaptic strength $g$.

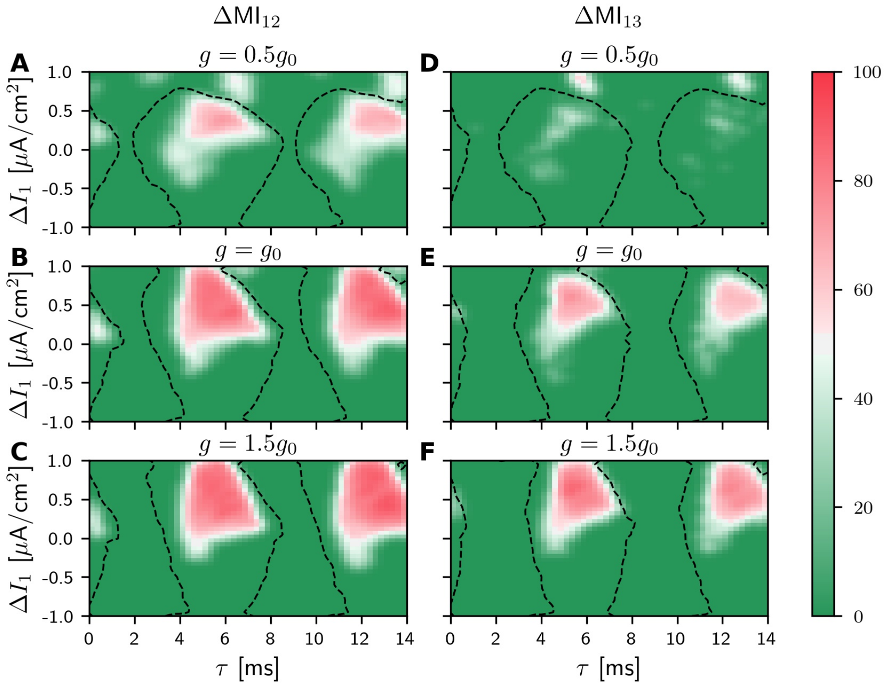


**Figure S7. V-motif**: Difference $\Delta \mathrm{MI}_{ij}$ between the firing rates of population 1 and 2 (A-C) and population 1 and 3 (D-F) when a slow modulation is injected as a function of the delay $\tau$ and the frequency mismatch $\Delta I$ in population 1 for different values of the synaptic strength $g$.

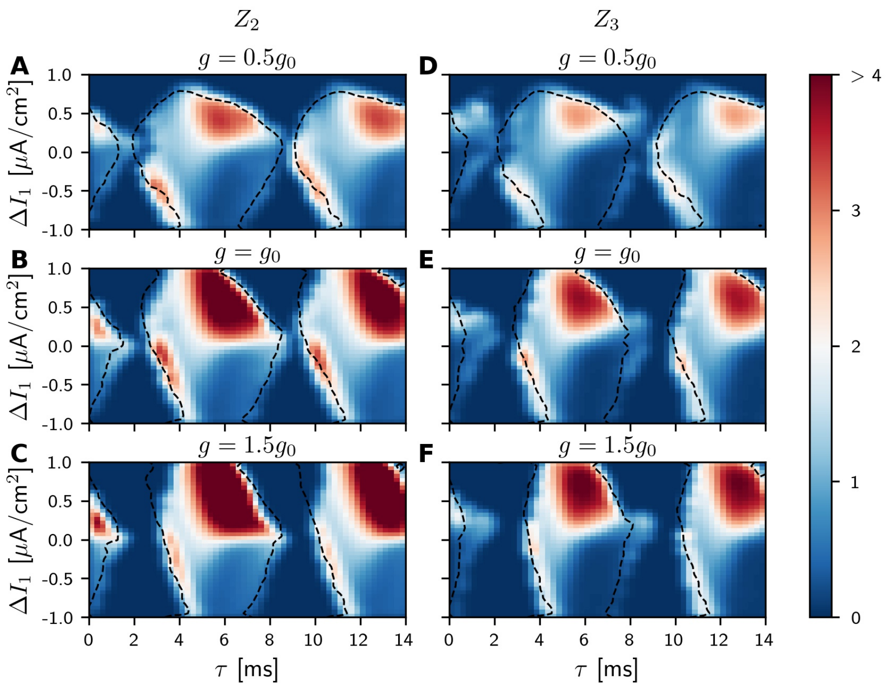


**Figure S8. V-motif**: Integral of the absolute value of the nPRC of the population 2 (A-C) and population 3 (D-F) when a fast signal is injectd as a function of the delay $\tau$ and the frequency mismatch $\Delta I$ in population 1 for different values of the synaptic strength $g$.

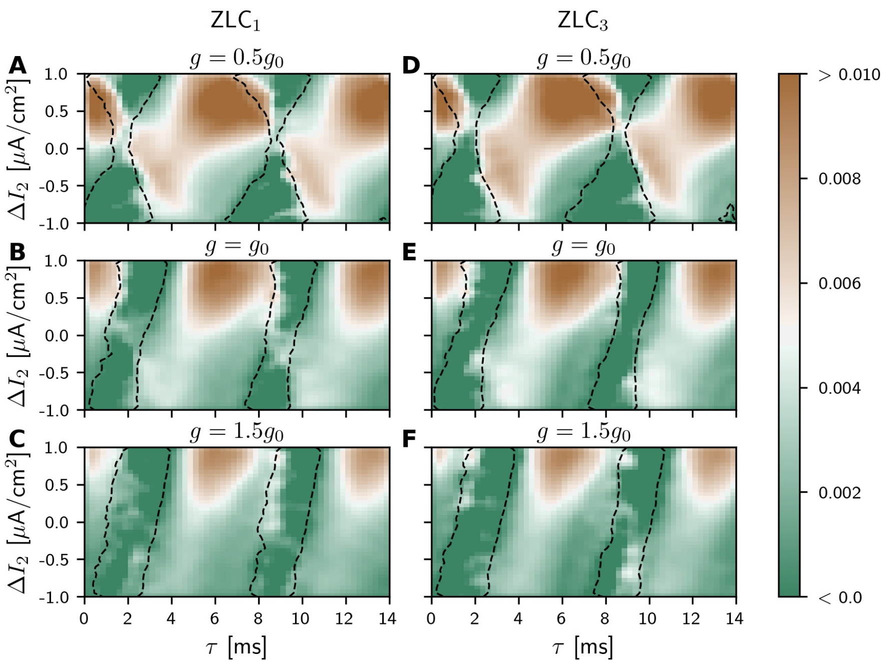


**Figure S9. V-motif**: Zero-lag cross covariance (ZLC) of the firing rates of the first (A-C) and third (D-F) population with the slow modulation injected as function of the delay $\tau$ and the frequency mismatch $\Delta I$ in population 2 for different values of the synaptic strength $g$.

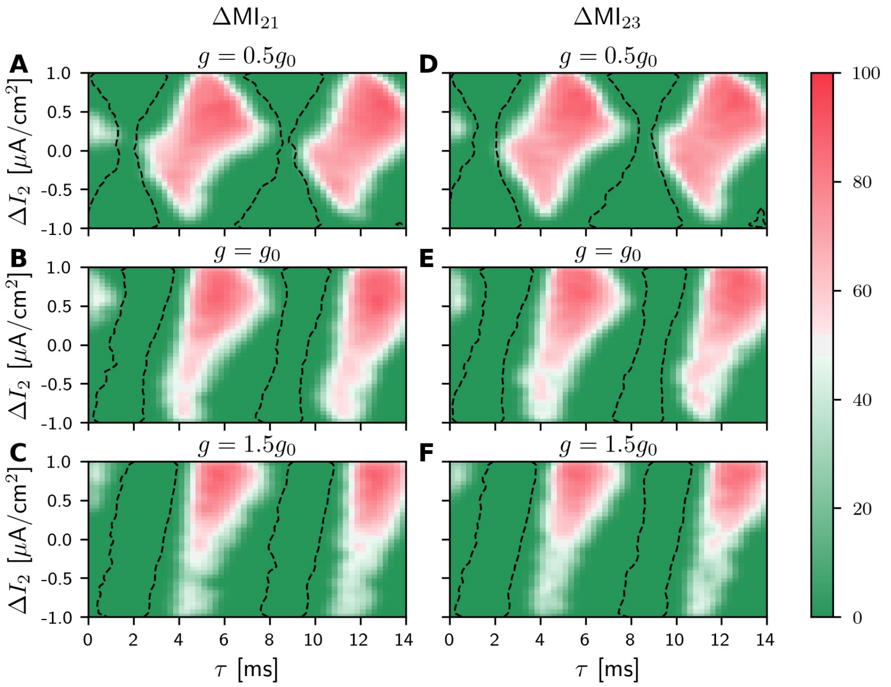


**Figure S10. V-motif**: Difference $\Delta\mathrm{MI}_{ij}$ between the firing rates of population 2 and 1 (A-C) and population 2 and 3 (D-F) when a slow modulation is injected as a function of the delay $\tau$ and the frequency mismatch $\Delta I$ in population 2 for different values of the synaptic strength $g$.

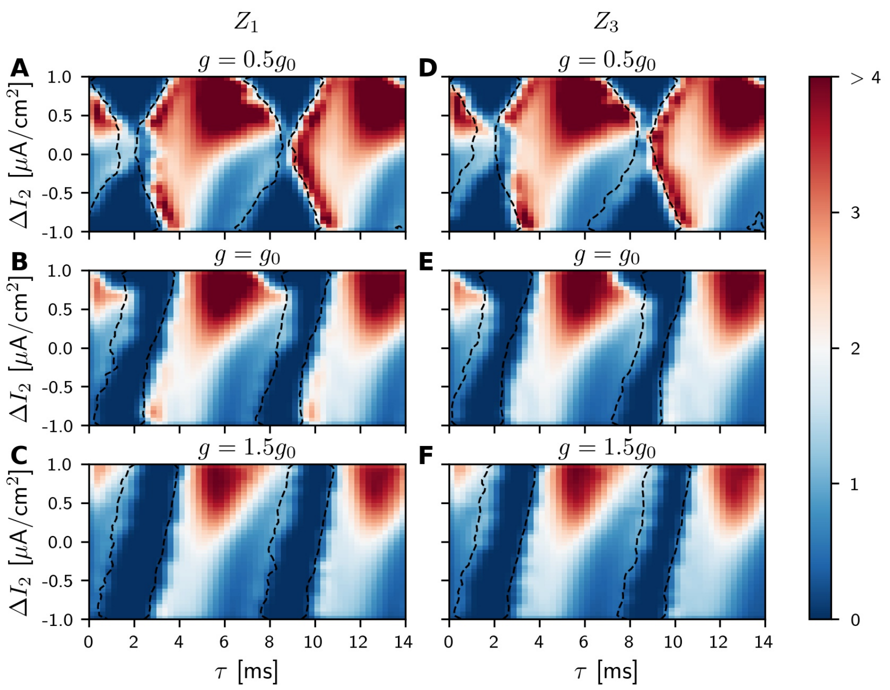


**Figure S11. V-motif**: Integral of the absolute value fo the nPRC of the population 1 (A-C) and population 3 (D-F) when a fast modulation is injected as a function of the delay $\tau$ and the frequency mismatch $\Delta I$ in population 2 for different values of the synaptic strength $g$.

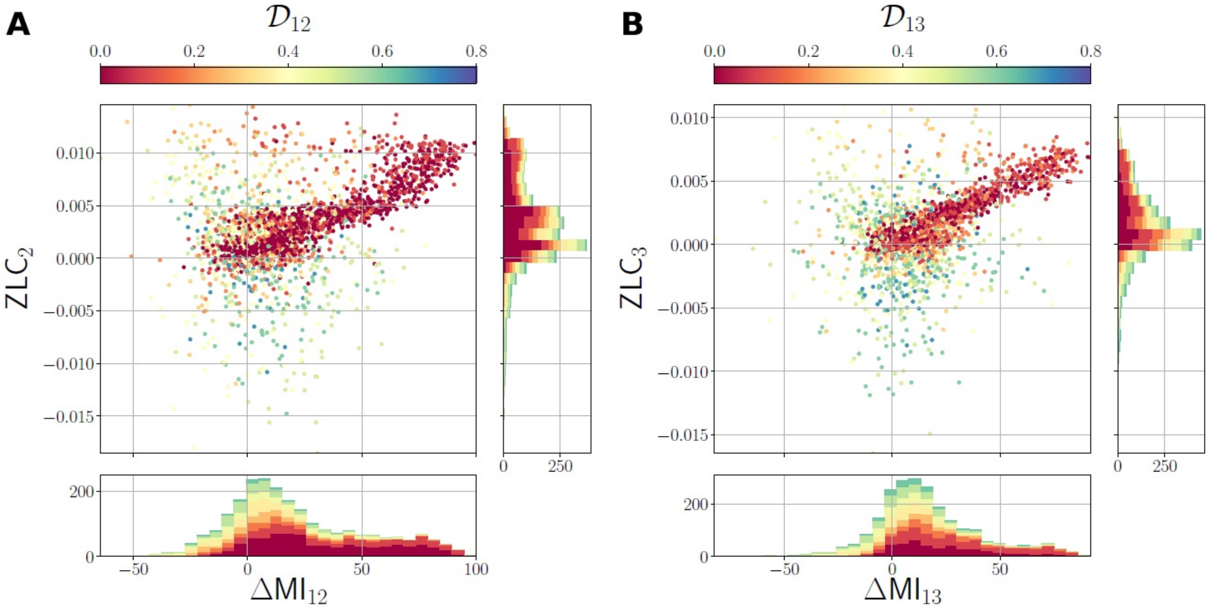


**Figure S12. V-motif**: Histograms of ZLC$_i$ and $\Delta$MI$_{1i}$ ($i$=2,3) as a function of the phase-locking index $\mathcal{D}$, for $g = g_0$. From the central plot, one can see how both measurements are non-linearly correlated when the phase-locking index is proximal to zero (perfect locking). Furthermore, high values of both measurements coincide with almost zero values of $\mathcal{D}$, implying that a better communication is achieved when the sender and receptor exhibit a constant phase difference relation.

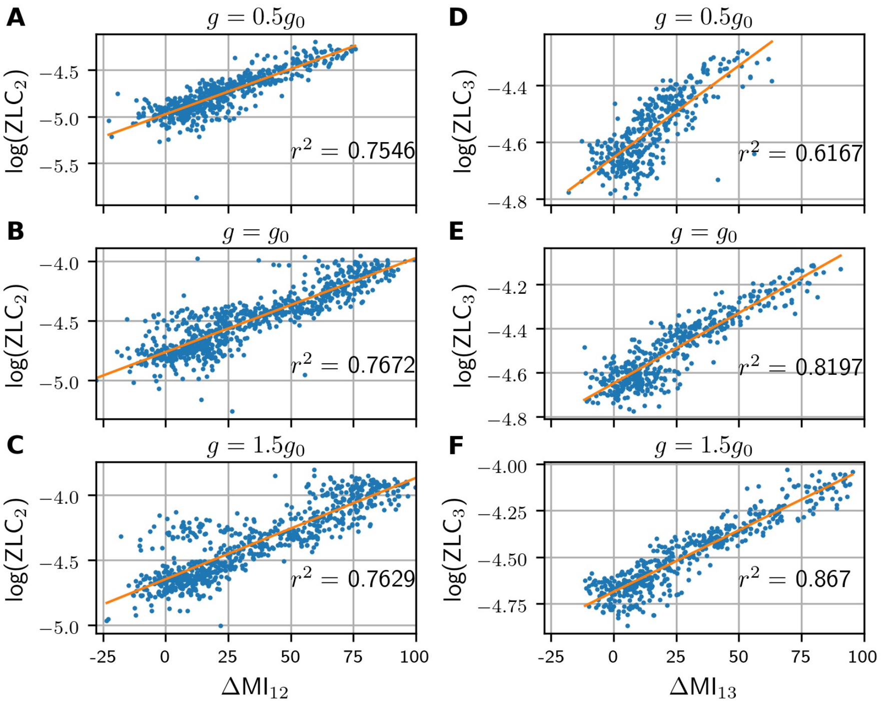


**Figure S13. V-motif**: R-squared regression of the natural logarithmic of $\mathrm{ZLC}_i$ and $\Delta\mathrm{MI}_{1i}$ ($i$ = 2,3) for values of the phase-locking index $\mathcal{D}$ lower than 0.25. To avoid indeterminations, a bias term has been added to $\mathrm{ZLC}_i$ values before computing the logarithmic. This confirms the relation observerd in S12 between these two measurement over the condition of $\mathcal{D}$ being proximal to zero.

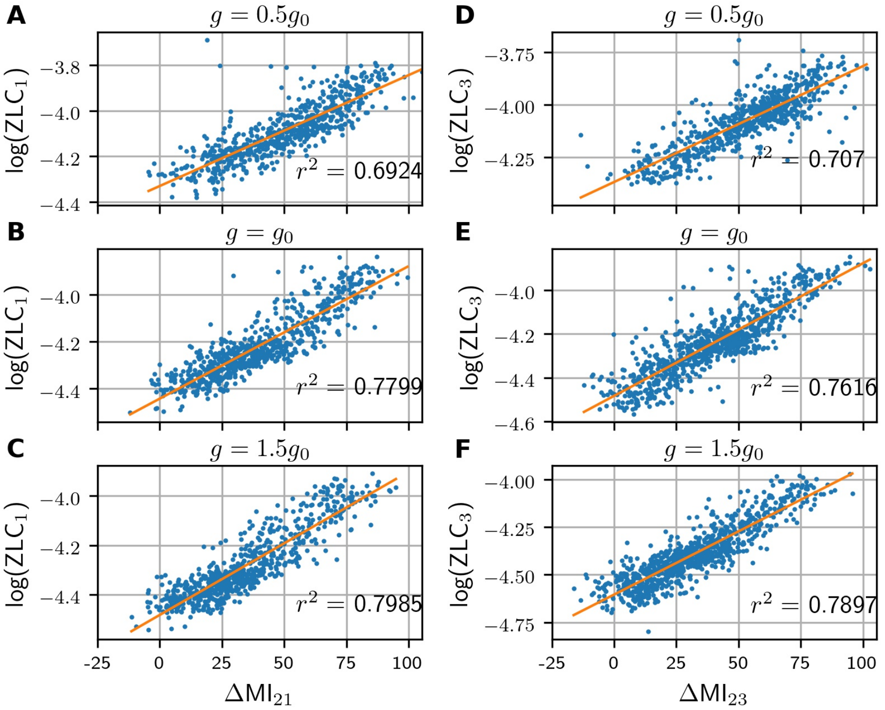


**Figure S14. V-motif**: R-squared regression of the natural logarithmic of $\mathrm{ZLC}_i$ and $\Delta\mathrm{MI}_{2i}$ ($i$ = 1,3) for values of the phase-locking index $\mathcal{D}$ lower than 0.25. To avoid indeterminations, a bias term has been added to $ZLC_i$ values before computing the logarithmic.

# 4 CIRCULAR MOTIF: SYNCHRONIZATION AND PHASE-LOCKING

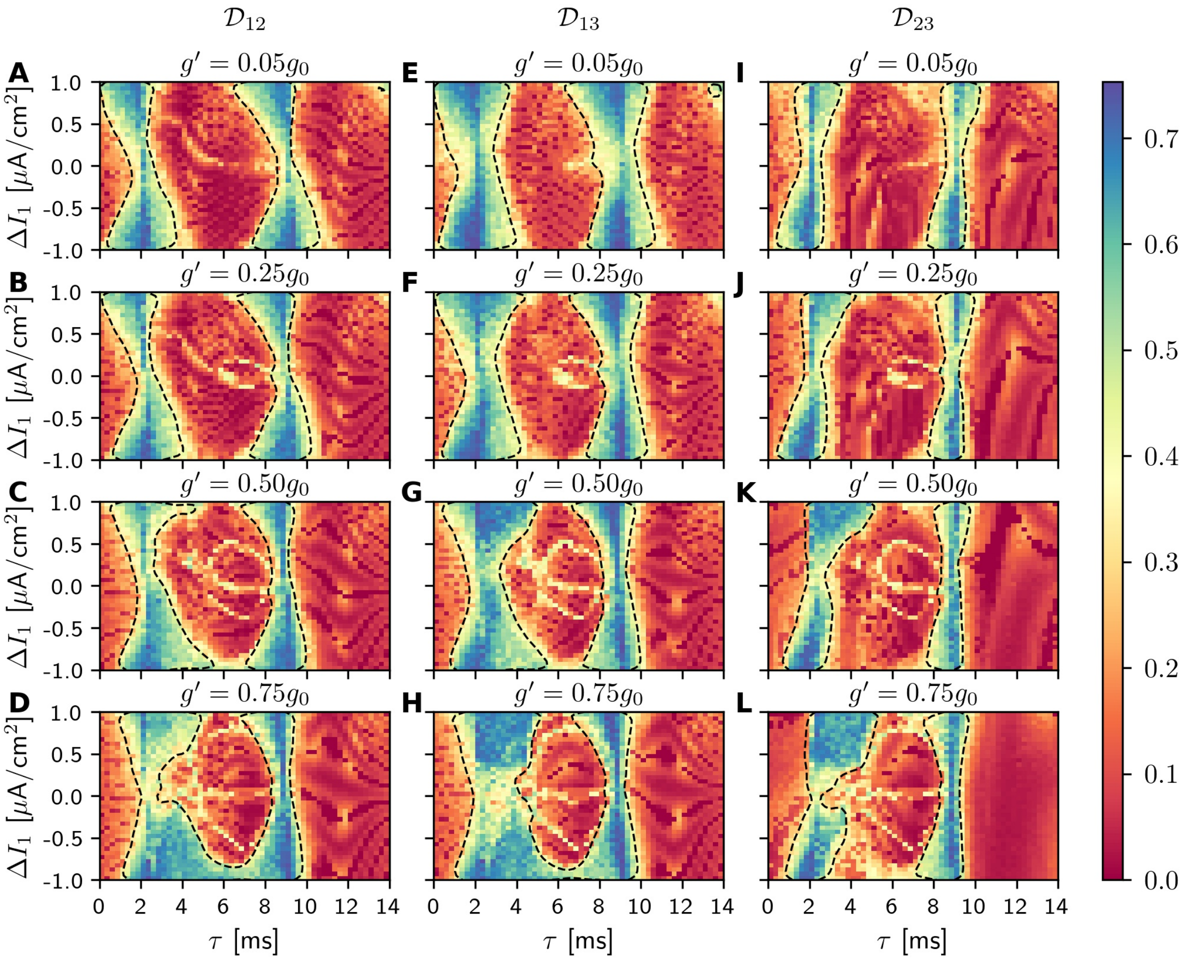


**Figure S15. Circular motif**: Phase locking between population 1 and 2 (A-D), population 1 and 3 (E-H), and population 2 and 3 (I-K) as a function of the delay $\tau$ and the frequency mismatch $\Delta I$ in population 1 for different values of the synaptic strength $g'$.

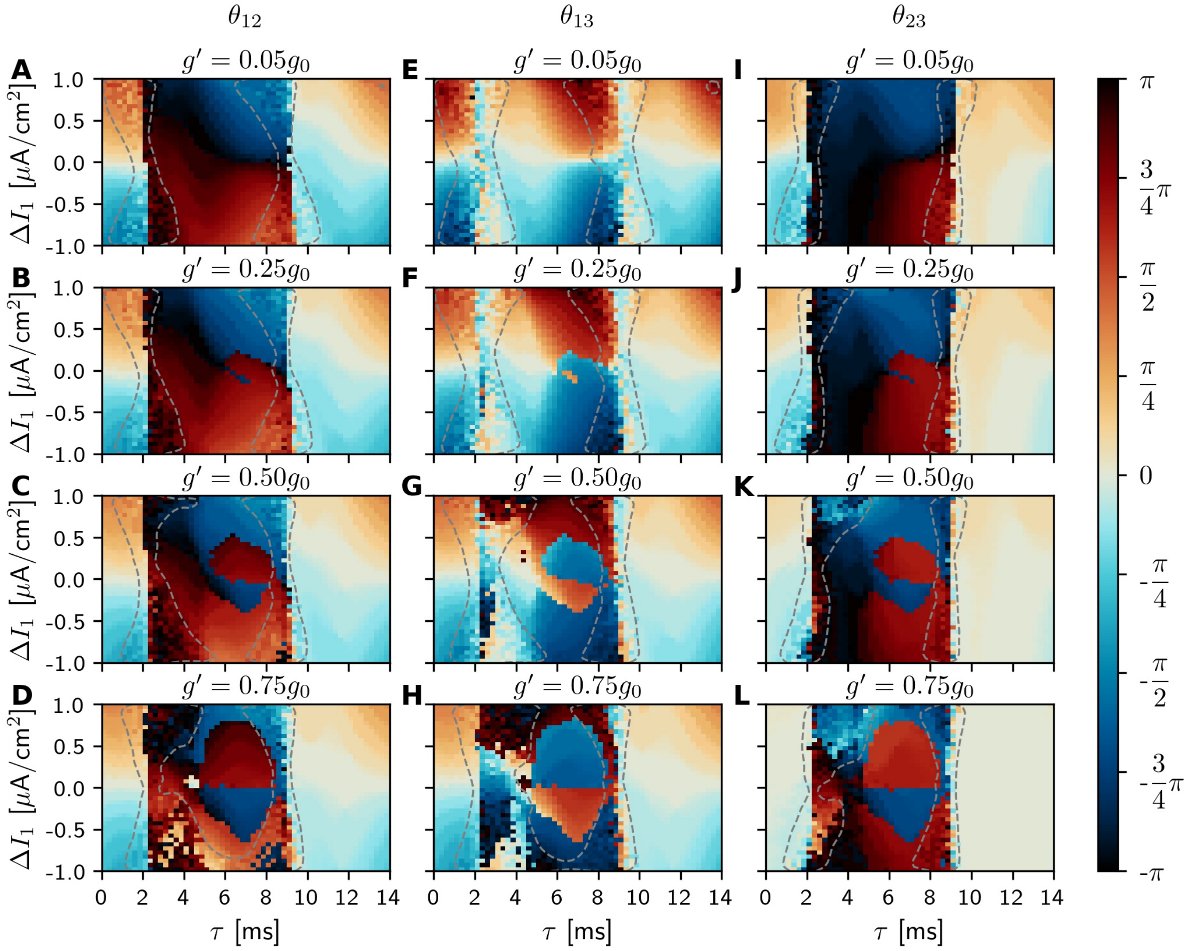


**Figure S16. Circular motif**: Phase difference between population 1 and 2 $\theta_{12}$ (A-D), population 1 and 3 $\theta_{13}$ (E-H), and population 2 and 3 $\theta_{23}$ (I-K) as a function of the delay $\tau$ and the frequency mismatch $\Delta I$ in population 1 for different values of the synaptic strength $g'$.

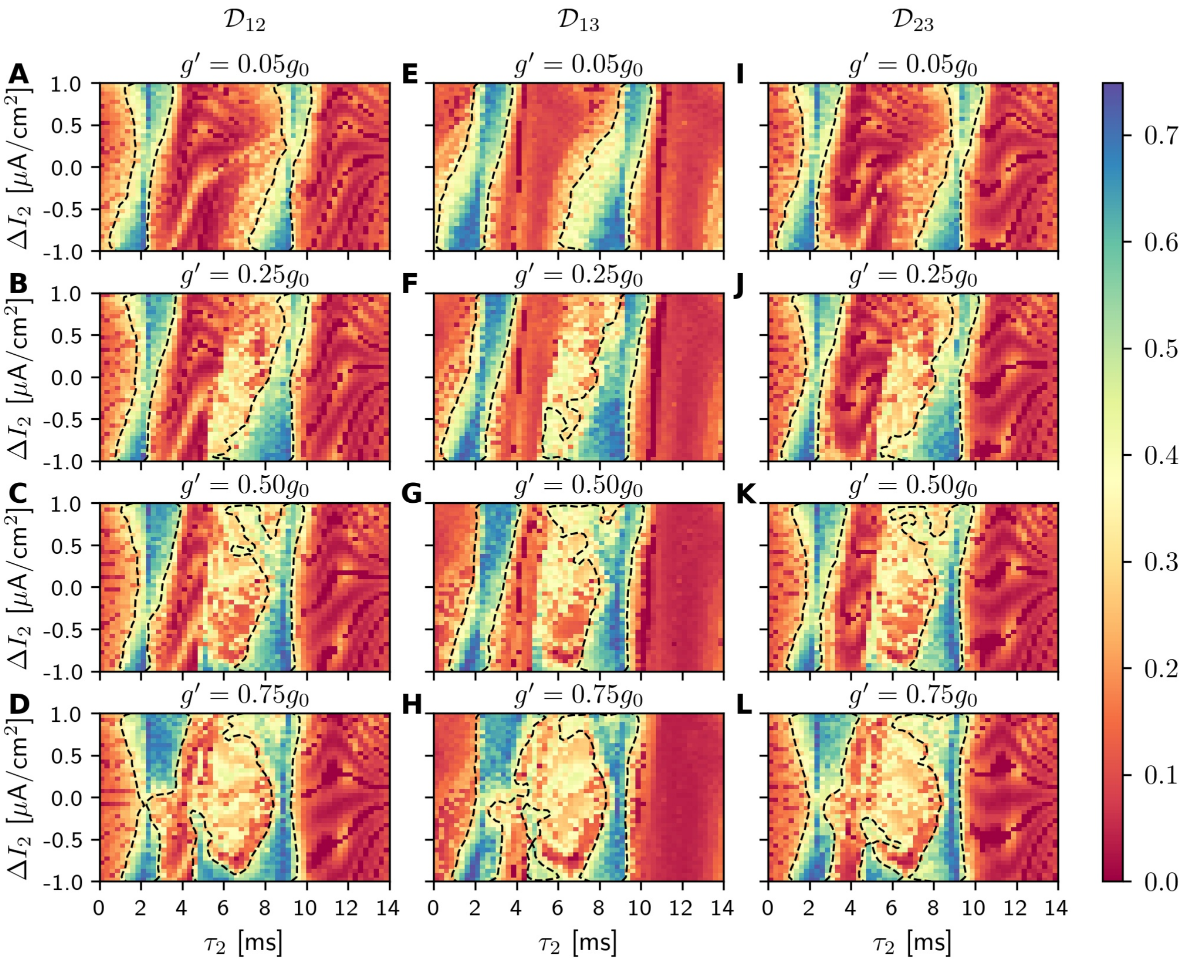


**Figure S17. Circular motif**: Phase locking between population 1 and 2 (A-D), population 1 and 3 (E-H), and population 2 and 3 (I-K) as a function of the delay $\tau$ and the frequency mismatch $\Delta I$ in population 2 for different values of the synaptic strength $g$.

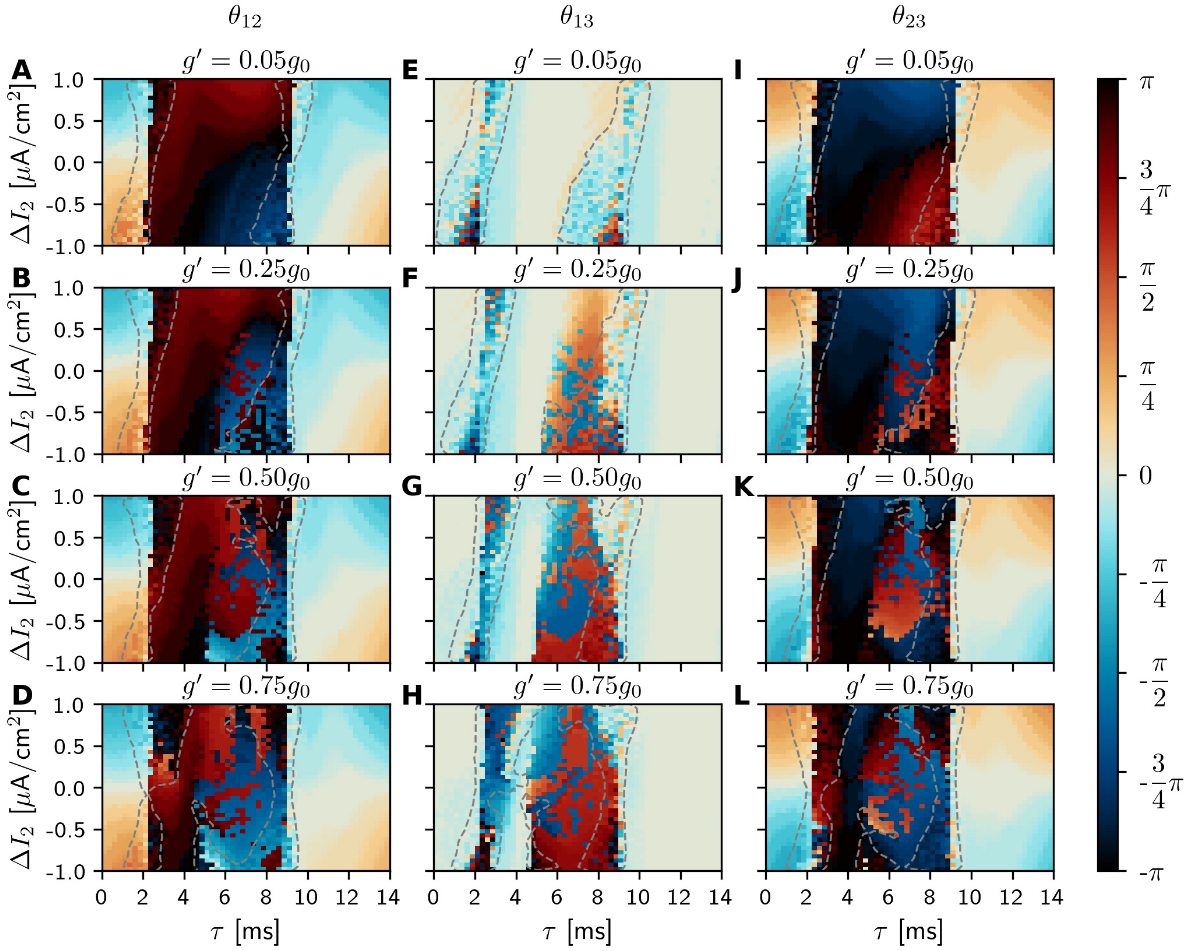


**Figure S18. Circular motif**: Phase difference between population 1 and 2 $\theta_{12}$ (A-D), population 1 and 3 $\theta_{13}$ (E-H),and population 2 and 3 $\theta_{23}$ (I-K) as a function of the delay $\tau$ and the frequency mismatch $\Delta I$ in population 2 for different values of the synaptic strength $g'$.

# 5 CIRCULAR MOTIF: INFORMATION TRANSMISSION

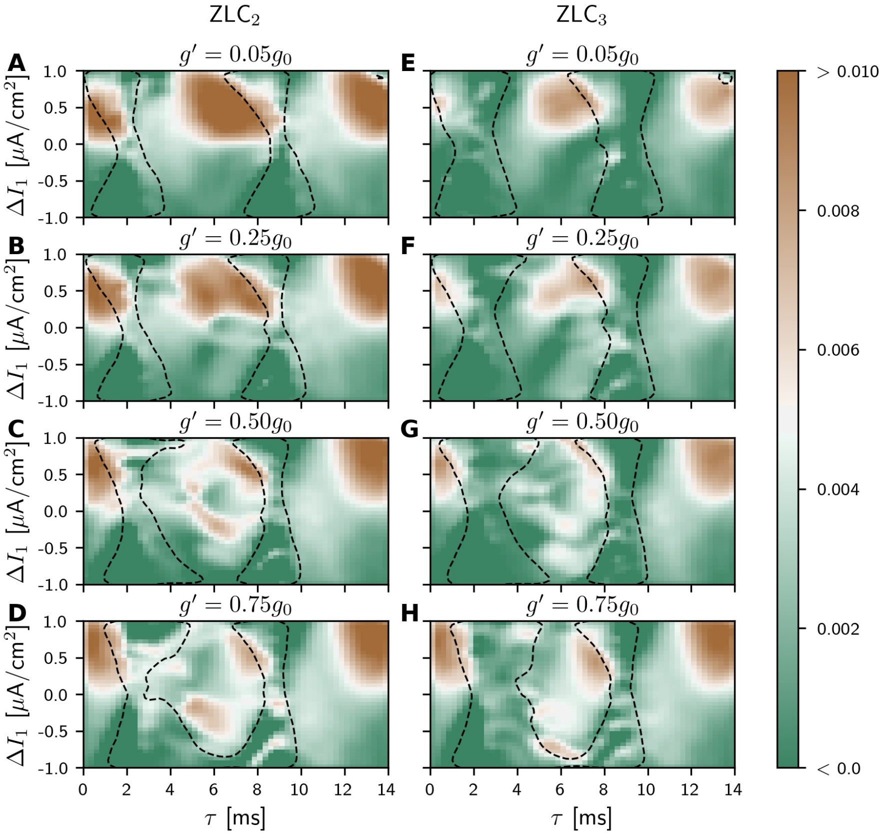


**Figure S19. Circular motif**: Zero-lag cross covariance (ZLC) of the firing rates of the first (A-D) and third (E-H) population with the slow modulation injected as function of the delay $\tau$ and the frequency mismatch $\Delta I$ in population 1 for different values of the synaptic strength $g'$.

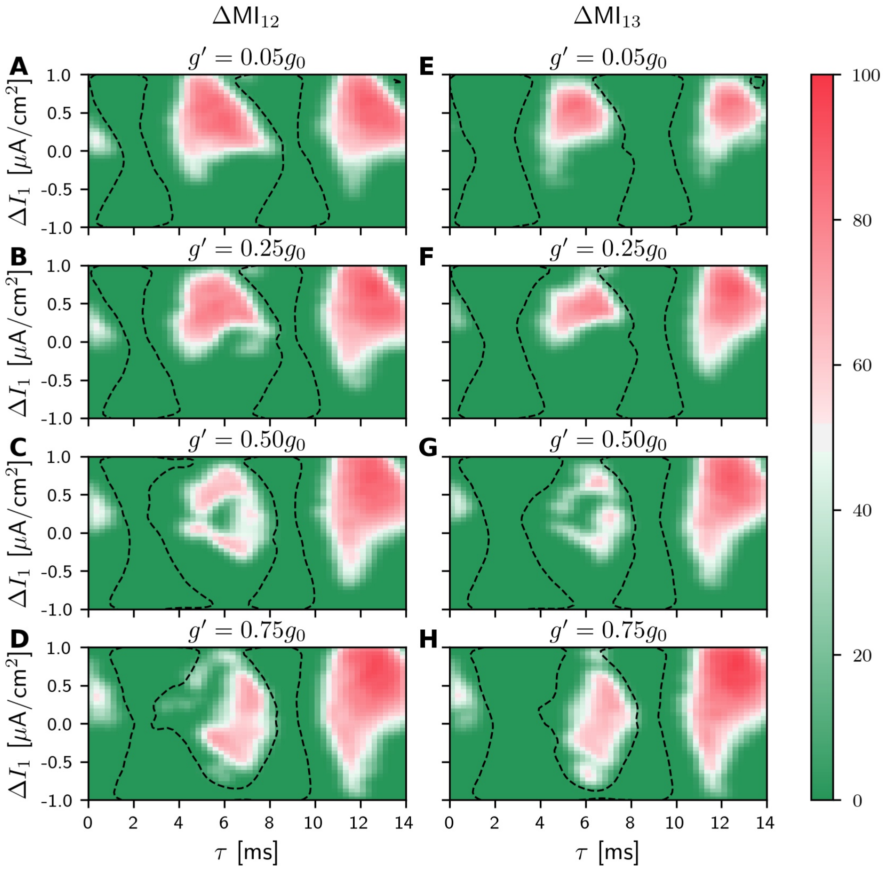


**Figure S20. Circular motif**: Difference $\Delta \mathrm{MI}_{ij}$ between the firing rates of population 1 and 2 (A-D) and population 1 and 3 (E-H) when a slow modulation is injected as a function of the delay $\tau$ and the frequency mismatch $\Delta I$ in population 1 for different values of the synaptic strength $g$.

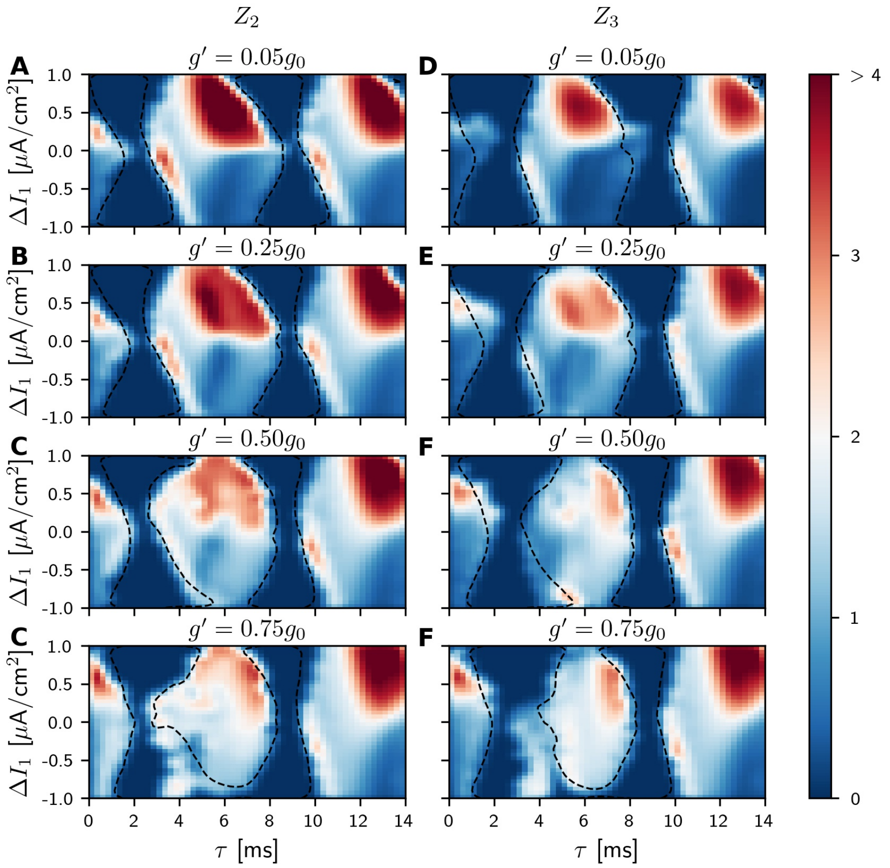


**Figure S21. Circular motif**: Integral of the absolute value of the nPRC of the population 2 (A-D) and population 3 (E-H) when a fast signal is injected as a function of the delay $\tau$ and the frequency mismatch $\Delta I$ in population 1 for different values of the synaptic strength $g$.

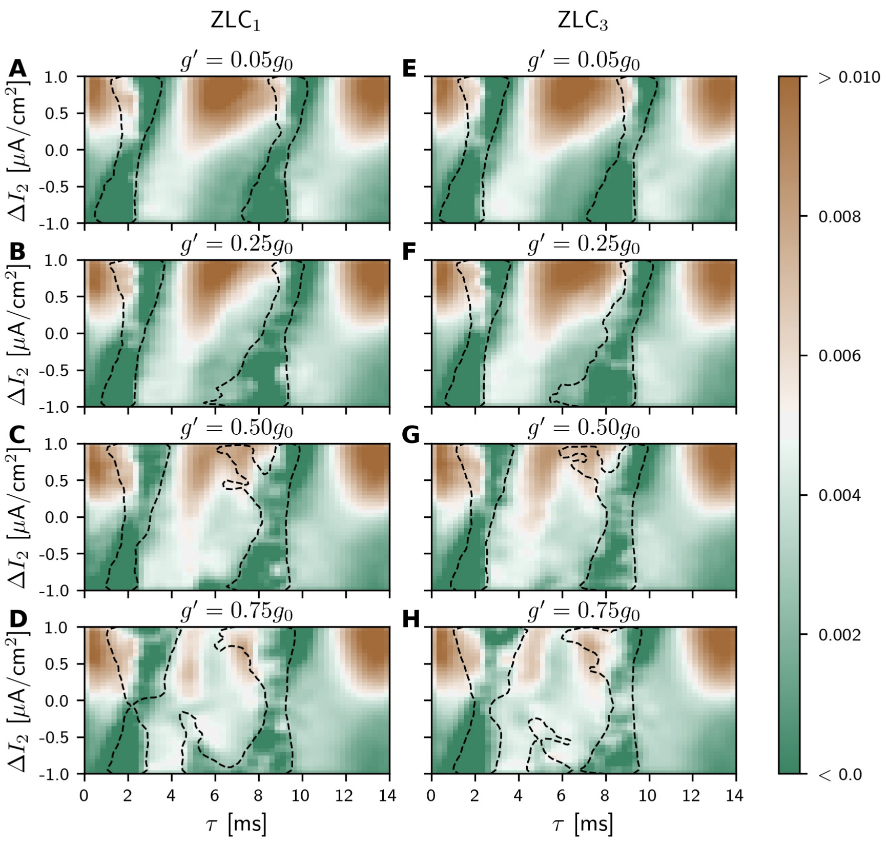


**Figure S22. Circular motif**: Zero-lag cross covariance (ZLC) of the firing rates of the first (A-D) and third (E-H) population with the slow modultion injected as function of the delay $\tau$ and the frequency mismatch $\Delta I$ in population 2 for different values of the synaptic strength $g'$.

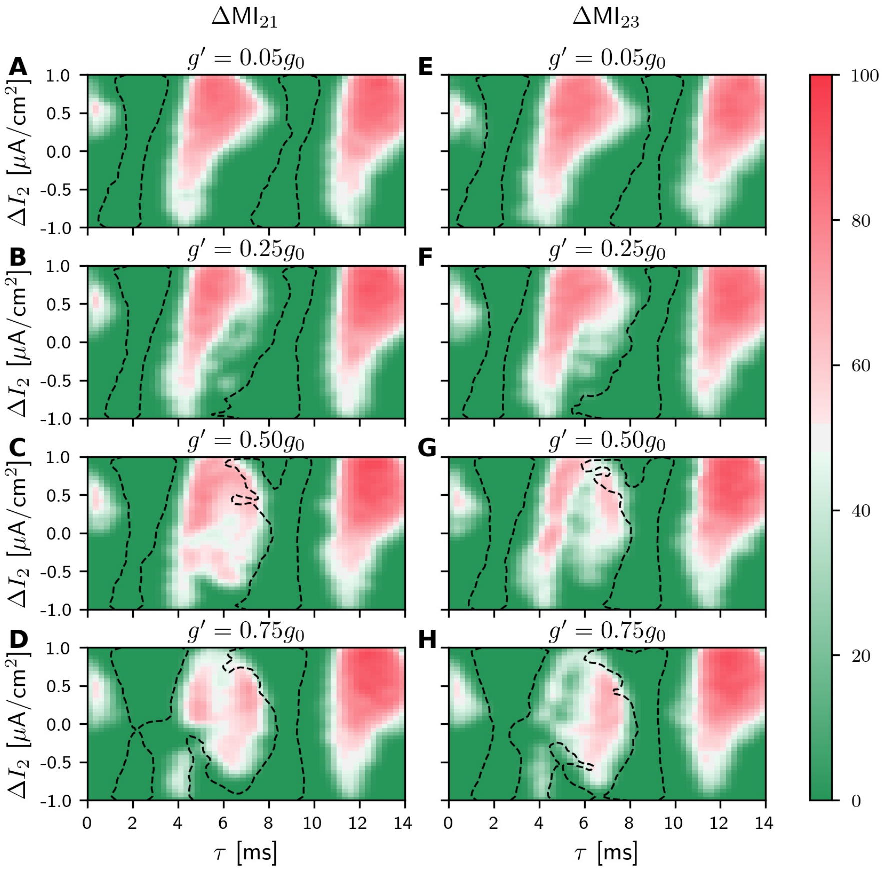


**Figure S23. Circular motif**: Difference $\Delta\mathrm{MI}_{ij}$ between the firing rates of population 2 and 1 (A-D) and population 2 and 3 (E-H) when a slow modulation is injected as a function of the delay $\tau$ and the frequency mismatch $\Delta I$ in population 2 for different values of the synaptic strength $g$.

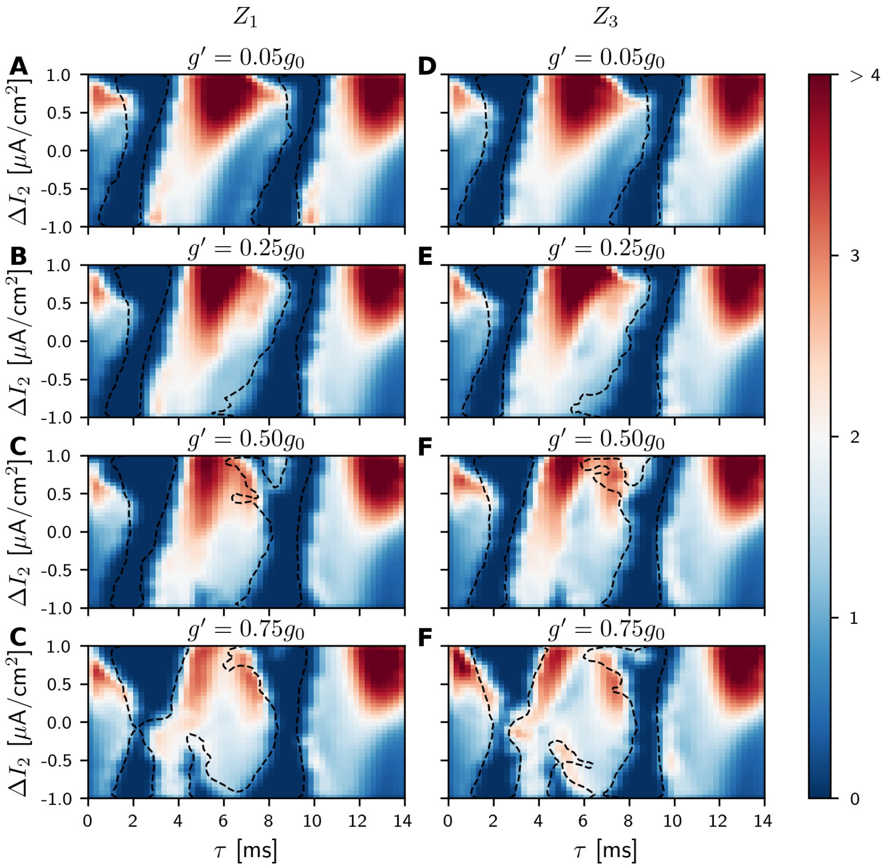


**Figure S24. Circular motif**: Integral of the absolute value fo the nPRC of the population 1 (A-D) and population 3 (E-H) when a fast signal is injected as a function of the delay $\tau$ and the frequency mismatch $\Delta I$ in population 2 for different values of the synaptic strength $g$.
